# Pediatric hospitalization profile in Australia: A longitudinal ecological study, 1998 to 2019

**DOI:** 10.1097/MD.0000000000047986

**Published:** 2026-03-13

**Authors:** Esra’ O. Taybeh, Alaa A. Alsharif, Abdallah Y. Naser

**Affiliations:** a Department of Applied Pharmaceutical Sciences and Clinical Pharmacy, Faculty of Pharmacy, Isra University, Amman, Jordan; b Department of Pharmacy Practice, College of Pharmacy, Princess Nourah bint Abdulrahman University, Riyadh, Saudi Arabia.

**Keywords:** admission, Australia, children, hospitalization, pediatrics

## Abstract

Examining pediatric hospitalization profile is important for healthcare planning and provision. The aim of this study was to identify the most common causes of hospitalization for the pediatric population in Australia between 1998 and 2019. This was an ecological study that examined the hospitalization profile for pediatric population in Australia using the National Hospital Morbidity Database. Between 1998 and 2019, there were 16,966,610 reported hospital admission episodes among the pediatric population in Australia. The number of annual admissions increased by 23.0%. Children who were admitted to the hospital for overnight-stay admissions comprised 55.9% of all admissions. Rates of same-day hospital admission among pediatrics increased by 22.1% [from 5596.7 (95% confidence interval [CI]: 5577.0–5616.4) in 1998 to 6832.1 (95% CI: 6812.3–6851.9) in 2019 per 100,000 persons, *P* ≤ .05]. Rates of overnight-stay hospital admission among pediatrics declined by 9.7%. Diseases of the respiratory system accounted for 15.1% of all hospital admissions. Hospital admission rates among females rose by 6.1% [from 13294.5 (95% CI: 13,252.8–13,336.2) in 1998 to 14,105.5 (95% CI: 14,066.2–14,144.7) in 2019 per 100,000 persons], compared to a 0.1% increase among males. While pediatric hospital admission counts increased substantially, the overall hospitalization rate remained largely stable. Important shifts were observed, including a rise in same-day admissions and higher admission rates among females. The predominance of respiratory conditions in young children and increasing hospitalizations in adolescents, particularly females, highlights the need for targeted strategies such as improved respiratory infection prevention, early parental guidance for acute illness, injury prevention programs, and expanded youth mental health services.

## 1. Introduction

Children comprise between a 3rd to almost half of the population in most countries.^[1]^ In 2020, an estimated 4.7 million children aged 0 to 14 and 1.5 million aged 15 to 19 years lived in Australia, making up 24.6% of the whole population.^[2]^ It is therefore important to understand the health care utilization patterns among pediatrics patients for the proper allocation of resources to improve pediatric health.

Children’s visits to emergency departments (EDs) and hospital admissions have increased noticeably in recent years.^[3,4]^ Previous studies suggest that the use of hospital services by children may be influenced by multiple factors. For example, the accessibility and availability of primary care, including after-hours general practitioner (GP) services, may shape patterns of hospital utilization.^[5]^ When children experience minor illnesses, parents are often expected to manage the illnesses on their own and seek medical help only if they feel they can no longer manage; however, this can sometimes result in hospital attendances for conditions that could have been managed elsewhere.^[6]^ In addition, limited access to healthcare, language barriers, or time constrains may lead parents to rely on alternative sources of information, such as social media or the internet, which has been suggested to heighten parental anxiety^[7]^ and potentially contribute to earlier healthcare-seeking behaviors.^[6]^

Regardless of the cause, admission to hospital is an undesirable option for both children and their parents since it is associated with family life interruption, increased emotional distress, exposure to nosocomial consequences, and significant cost implications.^[8]^

While previous scholars have focused on the increased hospital admissions of specific illnesses or within specific areas,^[9–11]^ it remains unclear whether the rise in all hospital admissions is uniform across all children and diseases. This gap highlights the need for research that marks trends in all-cause pediatrics hospitalizations that are disaggregated by age group, sex, or illnesses.

To address this gap, the present study examined nationally representative hospital admission data spanning 21 years (1998–2019) to describe temporal trends and the leading causes of pediatric hospitalization in Australia. Understanding temporal trends in pediatrics care and identifying the most common causes of hospitalization can inform healthcare initiatives aimed at reducing pediatrics hospitalization rates.

The significance of this study lies in its potential to inform healthcare policies and programs that can improve the health outcomes of children in Australia. The findings of the present study can also contribute to the existing literature on childhood hospitalization and serve as a basis for further research on this topic.

## 2. Methods

### 2.1. Study design

This was an ecological study that examined the hospitalization profile for pediatric population (aged below 19 years) in Australia between 1998 and 2019.

### 2.2. Data sources

### 2.3. The National Hospital Morbidity database

Data for this study was collected from the National Hospitals Data Collection in Australia.^[12]^ The data include information about the patients’ diagnoses, external causes of damage and poisoning, hospital stays, treatments, and demographics. The National Minimum Data Set includes care episodes for patients admitted to hospitals from all institutions.

Each patient must be given a status showing whether they were admitted on an emergency or elective basis, via a waitlist, through the ED, or directly as an emergency. If a patient has an illness or injury that needs evaluation and treatment within 24 hours, they are admitted on an emergency basis. Emergency patients may be admitted straight to a specialized area or through the ED (i.e., a critically ill patient who arrives at a hospital ED via an ambulance and is taken directly to the ICU). If a patient has a condition that does not require evaluation and treatment within 24 hours, they are admitted on an elective basis. Elective patients may be admitted either through the waitlist (i.e., after waiting 3 months for elective surgery) or without being placed in the queue (i.e., a patient admitted for a scheduled cesarean section).^[13]^

### 2.4. Australian Bureau of Statistics

Between 1998 and 2019, mid-year population data were gathered using the Australian Bureau of Statistics.^[14]^ Population data were gathered from the historical, national, state, and territorial populations data.^[15,16]^

### 2.5. Study population

This study collected data on all private and public hospitalizations for pediatric population in Australia from 1998 to 2019.^[17]^

### 2.6. Statistical analysis

All analyses were performed using SPSS version 27. (IBM Corp, Armonk) and Joinpoint Regression Software, version 5.4.0 (National Cancer Institute, Bethesda). Hospitalization rates with 95% confidence intervals (CIs) were calculated using the number of hospitalization episodes divided by the mid-year population using a normal (Wald) approximation. Trends over time were assessed using Joinpoint regression, which identifies points where significant changes in trends occur and calculates the annual percent change (APC) with associated CIs. Rates were aged standardized using the direct method to account for demographic shifts over the study period. The Australian mid-year population was the reference population. Premutation tests within Joinpoint were used to determine statistical significance of trends changes. Benjamini–Hochberg false discovery rate procedure (*q* = 0.05) was used to control for multiple testing across ICD chapters. To assess the variation in hospitalization rates between 1998 and 2019 we used the Pearson chi-square test for independence.

### 2.7. Ethical approval

This study used de-identified data and was considered exempt from human protection oversight by the institutional review board.

## 3. Results

### 3.1. Trends in total hospital admission among the pediatric population

Between 1998 and 2019, there were 16,966,610 reported hospital admission episodes among the pediatric population in Australia. From 739,022 in 1998 to 906,575 in 2019, the annual total number increased by 23.0%, representing a 2.9% increase in hospital admission rates [14,149.5 (95% CI: 14,119.6–14,179.4) in 1998 to 14,557.9 (95% CI: 14,530.2–14,585.6) in 2019 per 100,000 people, *P* ≥ .05].

**Figure 1.**
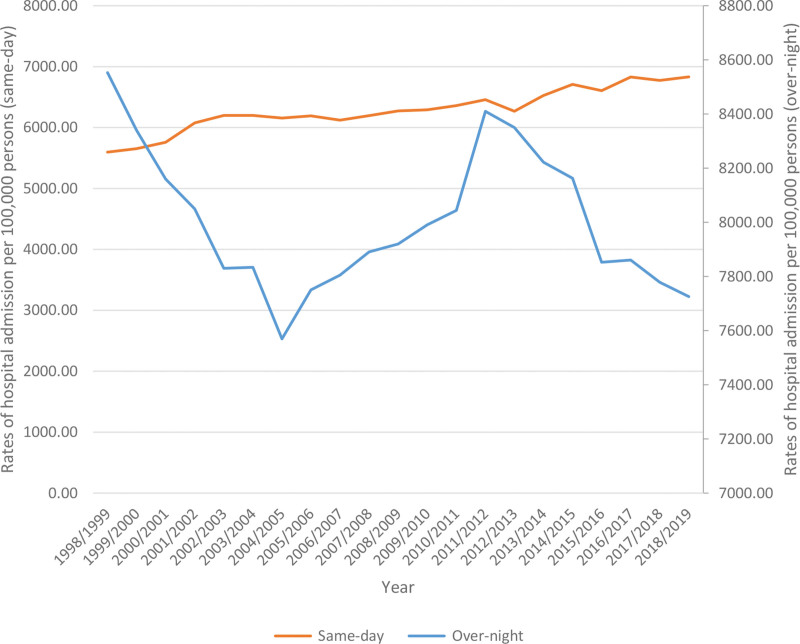
Rates of same-day and overnight-stay hospital admission among pediatrics population in Australia between 1998 and 2019.

Children who were admitted to the hospital; for overnight-stay admissions, comprised 55.9% of all pediatric admissions, and 44.1% of those were same-day admissions. Rates of same-day hospital admission among pediatrics increased by 22.1% [from 5596.7 (95% CI: 5577.0–5616.4) in 1998 to 6832.1 (95% CI: 6812.3–6851.9) in 2019 per 100,000 persons, *P* ≤ .05]. Rates of overnight-stay hospital admission among pediatrics declined by 9.7% [from 8552.7 (95% CI: 8528.8–8576.7) in 1998 to 7725.8 (95% CI: 7704.8–7746.8) in 2019 per 100,000 persons, *P* ≤ .05] (Fig. 1).

Hospital admission rates stratified by age and gender are presented separately in the supplementary file for same-day and overnight-stay hospital admissions (Figures S1 and S2, Supplemental Digital Content, https://links.lww.com/MD/R523). Diseases of the respiratory system accounted for 15.1% of all hospital admissions among pediatrics, making them the most common cause of hospital admission. Injury, poisoning, and certain other consequences of external causes ranked second at 13.4%, followed by diseases of the digestive system ranked third at 12.1% (Table 1).

**Table 1:** Percentage of hospital admission among pediatrics population from the total number of admissions.

ICD code	Description	Percentage from total number of admissions
J00–J99	“Diseases of the respiratory system (acute upper respiratory infections, influenza and pneumonia, other acute lower respiratory infections, other diseases of upper respiratory tract, chronic lower respiratory diseases, lung diseases due to external agents, other respiratory diseases principally affecting the interstitium, suppurative and necrotic conditions of the lower respiratory tract, other diseases of the pleura, and intraoperative and postprocedural complications, and disorders of respiratory system)”	15.1%
S00–T98	“Injury, poisoning, and certain other consequences of external causes (injuries to the head, injuries to the neck, injuries to the thorax, injuries to the abdomen, lower back, lumbar spine, pelvis and external genitals, injuries to the shoulder and upper arm, injuries to the elbow and forearm, injuries to the wrist, hand and fingers, injuries to the hip and thigh, injuries to the knee and lower leg, injuries to the ankle and foot, injuries involving multiple body regions, injury of unspecified body region, effects of foreign body entering through natural orifice, burns and corrosions of external body surface, specified by site, burns and corrosions confined to eye and internal organs, burns and corrosions of multiple and unspecified body regions, frostbite, poisoning by, adverse effect of and underdosing of drugs, medicaments and biological substances, toxic effects of substances chiefly nonmedicinal as to source, other and unspecified effects of external causes, certain early complications of trauma, and complications of surgical and medical care)”	13.4%
K00–K93	“Diseases of the digestive system (diseases of oral cavity and salivary glands, diseases of esophagus, stomach and duodenum, diseases of appendix, hernia, noninfective enteritis and colitis, other diseases of intestines, diseases of peritoneum and retroperitoneum, diseases of liver, and disorders of gallbladder, biliary tract and pancreas)”	12.1%
R00–R99	“Symptoms, signs and abnormal clinical and laboratory findings (symptoms and signs involving the circulatory and respiratory systems, symptoms and signs involving the digestive system and abdomen, symptoms and signs involving the skin and subcutaneous tissue, symptoms and signs involving the nervous and musculoskeletal systems, symptoms and signs involving the genitourinary system, symptoms and signs involving cognition, perception, emotional state and behavior, symptoms and signs involving speech and voice, general symptoms and signs, abnormal findings on examination of blood, without diagnosis, abnormal findings on examination of urine, without diagnosis, abnormal findings on examination of other body fluids, substances and tissues, without diagnosis, abnormal findings on diagnostic imaging and in function studies, without diagnosis, abnormal tumor markers, and ill-defined and unknown cause of mortality)”	7.4%
Z00–Z99	“Factors influencing health status and contact with health services (persons encountering health services for examinations, genetic carrier and genetic susceptibility to disease, resistance to antimicrobial drugs, estrogen receptor status, retained foreign body fragments, hormone sensitivity malignancy status, persons with potential health hazards related to communicable diseases, persons encountering health services in circumstances related to reproduction, encounters for other specific health care, persons with potential health hazards related to socioeconomic and psychosocial circumstances, do not resuscitate status, blood type, body mass index (BMI), persons encountering health services in other circumstances, and persons with potential health hazards related to family and personal history and certain conditions influencing health status)”	7.4%
P00–P96	“Certain conditions originating in the perinatal period (newborn affected by maternal factors and by complications of pregnancy, labor, and delivery, disorders of newborn related to length of gestation and fetal growth, abnormal findings on neonatal screening, birth trauma, respiratory and cardiovascular disorders specific to the perinatal period, infections specific to the perinatal period, hemorrhagic and hematological disorders of newborn, transitory endocrine and metabolic disorders specific to newborn, digestive system disorders of newborn, conditions involving the integument and temperature regulation of newborn, and other problems with newborn)”	7.2%
A00–B99	“Certain infectious and parasitic diseases (intestinal infectious diseases, tuberculosis, certain zoonotic bacterial diseases, other bacterial diseases, infections with a predominantly sexual mode of transmission, other spirochetal diseases, other diseases caused by chlamydiae, rickettsioses, viral and prion infections of the central nervous system, arthropod-borne viral fevers and viral hemorrhagic fevers, viral infections characterized by skin and mucous membrane lesions, other human herpesviruses, viral hepatitis, human immunodeficiency virus [HIV] disease, other viral diseases, mycoses, protozoal diseases, helminthiases, pediculosis, acariasis and other infestations, sequelae of infectious and parasitic diseases, and bacterial and viral infectious agents)”	5.0%
H60–H95	“Diseases of the ear and mastoid process (diseases of external ear, diseases of middle ear and mastoid, diseases of inner ear, other disorders of ear, and intraoperative and postprocedural complications and disorders of ear and mastoid process)”	4.1%
G00–G99	“Diseases of the nervous system (inflammatory diseases of the central nervous system, systemic atrophies primarily affecting the central nervous system, extrapyramidal and movement disorders, other degenerative diseases of the nervous system, demyelinating diseases of the central nervous system, episodic and paroxysmal disorders, nerve, nerve root and plexus disorders, polyneuropathies and other disorders of the peripheral nervous system, diseases of myoneural junction and muscle, and cerebral palsy and other paralytic syndromes)”	3.4%
N00–N99	“Diseases of the genitourinary system (glomerular diseases, renal tubulo-interstitial diseases, acute kidney failure and chronic kidney disease, urolithiasis, other disorders of kidney and ureter, other diseases of the urinary system, diseases of male genital organs, disorders of breast, inflammatory diseases of female pelvic organs, noninflammatory disorders of female genital tract, and intraoperative and postprocedural complications and disorders of genitourinary system)”	3.4%
F00–F99	“Mental and behavioral disorders (mental disorders due to known physiological conditions, mental and behavioral disorders due to psychoactive substance use, schizophrenia, schizotypal, delusional, and other non-mood psychotic disorders, mood [affective] disorders, anxiety, dissociative, stress-related, somatoform and other nonpsychotic mental disorders, behavioral syndromes associated with physiological disturbances and physical factors, disorders of adult personality and behavior, intellectual disabilities, pervasive and specific developmental disorders, and behavioral and emotional disorders with onset usually occurring in childhood and adolescence)”	3.3%
O00–O99	“Pregnancy, childbirth and the puerperium (pregnancy with abortive outcome, supervision of high risk pregnancy, edema, proteinuria and hypertensive disorders in pregnancy, childbirth and the puerperium, other maternal disorders predominantly related to pregnancy, aternal care related to the fetus and amniotic cavity and possible delivery problems, complications of labor and delivery, encounter for delivery, and complications predominantly related to the puerperium, and Other obstetric conditions)”	3.2%
Q00–Q99	“Congenital malformations, deformations and chromosomal abnormalities (congenital malformations of the nervous system, congenital malformations of eye, ear, face and neck, congenital malformations of the circulatory system, congenital malformations of the respiratory system, cleft lip and cleft palate, other congenital malformations of the digestive system, congenital malformations of genital organs, congenital malformations of the urinary system, congenital malformations and deformations of the musculoskeletal system, other congenital malformations, and chromosomal abnormalities)”	3.2%
M00–M99	“Diseases of the musculoskeletal system and connective tissue (infectious arthropathies, autoinflammatory syndromes, inflammatory polyarthropathies, osteoarthritis, other joint disorders, dentofacial anomalies [including malocclusion] and other disorders of jaw, systemic connective tissue disorders, deforming dorsopathies, spondylopathies, other dorsopathies, disorders of muscles, disorders of synovium and tendon, other soft tissue disorders, disorders of bone density and structure, other osteopathies, chondropathies, other disorders of the musculoskeletal system and connective tissue, intraoperative and postprocedural complications and disorders of musculoskeletal system, periprosthetic fracture around internal prosthetic joint, and biomechanical lesions)”	2.8%
L00–L99	“Diseases of the skin and subcutaneous tissue (infections of the skin and subcutaneous tissue, bullous disorders, dermatitis and eczema, papulosquamous disorders, urticaria and erythema, radiation-related disorders of the skin and subcutaneous tissue, disorders of skin appendages, and intraoperative and postprocedural complications of skin and subcutaneous tissue)”	2.7%
C00–D48	“Neoplasms (malignant neoplasms of lip, oral cavity and pharynx, malignant neoplasms of digestive organs, malignant neoplasms of respiratory and intrathoracic organs, malignant neoplasms of bone and articular cartilage, melanoma and other malignant neoplasms of skin, malignant neoplasms of mesothelial and soft tissue, malignant neoplasms of breast, malignant neoplasms of female genital organs, malignant neoplasms of male genital organs, malignant neoplasms of urinary tract, malignant neoplasms of eye, brain and other parts of central nervous system, malignant neoplasms of thyroid and other endocrine glands, malignant neoplasms of ill-defined, other secondary and unspecified sites, malignant neuroendocrine tumors, secondary neuroendocrine tumors, malignant neoplasms of lymphoid, hematopoietic and related tissue, in situ neoplasms, benign neoplasms, except benign neuroendocrine tumors, neoplasms of uncertain behavior, polycythemia vera and myelodysplastic syndromes, benign neuroendocrine tumors, and neoplasms of unspecified behavior)”	2.1%
E00–E89	“Endocrine, nutritional and metabolic diseases (disorders of thyroid gland, diabetes mellitus, other disorders of glucose regulation and pancreatic internal secretion, disorders of other endocrine glands, intraoperative complications of endocrine system, malnutrition, other nutritional deficiencies, overweight, obesity and other hyperalimentation, metabolic disorders, and postprocedural endocrine and metabolic complications and disorders)”	1.5%
D50–D89	“Diseases of the blood and blood-forming organs and certain disorders involving the immune mechanism (nutritional anemias, hemolytic anemias, aplastic and other anemias and other bone marrow failure syndromes, coagulation defects, purpura and other hemorrhagic conditions, other disorders of blood and blood-forming organs, intraoperative and postprocedural complications of the spleen, and certain disorders involving the immune mechanism)”	1.3%
H00–H59	“Diseases of the eye and adnexa (disorders of eyelid, lacrimal system and orbit, disorders of conjunctiva, disorders of sclera, cornea, iris and ciliary body, disorders of lens, disorders of choroid and retina, glaucoma, disorders of vitreous body and globe, disorders of optic nerve and visual pathways, disorders of ocular muscles, binocular movement, accommodation and refraction, visual disturbances and blindness, other disorders of eye and adnexa, and intraoperative and postprocedural complications and disorders of eye and adnexa)”	0.9%
I00–I99	“Diseases of the circulatory system (acute rheumatic fever, chronic rheumatic heart diseases, hypertensive diseases, ischemic heart diseases, pulmonary heart disease and diseases of pulmonary circulation, other forms of heart disease, cerebrovascular diseases, diseases of arteries, arterioles and capillaries, and diseases of veins, lymphatic vessels and lymph nodes)”	0.8%

The Joinpoint analysis revealed variable trends in hospital admission rate. For infectious diseases, the sharpest decline occurred between 2006 and 2009 (APC = –9.41%, 95% CI: –12.22 to –3.7, *P* < .001), while the overall trend (average annual percent change [AAPC] = –1.17%, 95% CI: –1.6 to –0.57, *P* < .001) indicated a significant decrease. Neoplasms showed a modest but significant long-term decline (AAPC = –1.09%, 95% CI: –1.48 to –0.8, *P* < .001), with the steepest drop between 2014 and 2018 (APC = –3.6%, *P* < .001). Endocrine disease rose overall (AAPC = 2.34%, 95% CI: 2.09–2.58, *P* < .001), especially between 2004 and 2009 (APC = 4.51%, *P* = .01) and 2015 and 2018 (APC = 4.12%, *P* < .001). Further details are provided in Table 2.

**Table 2:** Trends in hospital admission rates by disease category: Joinpoint regression analysis, 1998 to 2018.

Cohort	Range	APC (95% CI)	*P*-value	AAPC (95% CI)	*P*-value	Benjamini–Hochberg FDR *q*-values
Certain infectious	1998–2006	−0.42 (−1.53 to 2.44)	.74	−1.17 (−1.6 to −0.57)	<.001	0.00167
	2006–2009	−9.41 (−12.22 to −3.7)	<.001			
	2009–2018	1.06 (−0.21 to 4)	.09			
Neoplasms	1998–2014	−0.46 (−0.72 to −0.01)	.05	−1.09 (−1.48 to −0.8)	<.001	0.00167
	2014–2018	−3.6 (−7.38 to −1.74)	<.001			
Diseases of the blood	1998–2000	−6.27 (−8.44 to −0.61)	.02	0.01 (−0.21 to 0.47)	.94	0.94
	2000–2018	0.73 (0.49 to 1.17)	.01			
Diseases of endocrine system	1998–2004	2.41 (−0.42 to 3.42)	.08	2.34 (2.09 to 2.58)	<.001	0.00167
	2004–2009	4.51 (3.3 to 6.55)	.01			
	2009–2015	−0.36 (−2.65 to 0.43)	.27			
	2015–2018	4.12 (2.04 to 7.29)	<.001			
Mental disorders	1998–2004	0.03 (−1.16 to 3.29)	.75	−0.25 (−0.52 to 0.11)	.19	0.211
	2004–2007	−6.97 (−8.79 to −3.13)	.01			
	2007–2018	1.51 (0.93 to 2.32)	.01			
Diseases of the nervous system	1998–2014	3.83 (3.51 to 6.13)	<.001	3.34 (2.98 to 3.85)	<.001	0.00167
	2014–2018	1.37 (−2.75 to 3.31)	.37			
Diseases of the eye and adnexa	1998–2006	−2 (−2.65 to −1.61)	<.001	−1.14 (−1.3 to −1.03)	<.001	0.00167
	2006–2010	1.03 (−0.09 to 2.37)	.08			
	2010–2018	−1.36 (−1.99 to −1.02)	<.001			
Diseases of the ear and mastoid process	1998–2006	−3.55 (−4.49 to −2.9)	<.001	−1.39 (−1.63 to −1.19)	<.001	0.00167
	2006–2010	2.7 (0.71 to 5.08)	<.001			
	2010–2018	−1.21 (−2.32 to −0.67)	<.001			
Diseases of the circulatory system	1998–2002	−0.1 (−5.25 to 2.6)	.95	2.54 (2.13 to 3.02)	<.001	0.00167
	2002–2018	3.21 (2.88 to 4.40)	.01			
Diseases of the respiratory system	1998–2003	−3.20 (−9.19 to −0.43)	.02	−0.17 (−0.55 to 0.4)	.44	0.463
	2003–2018	0.87 (0.34 to 2.69)	.01			
Diseases of the digestive system	1998–2009	1.79 (1.5 to 2.16)	<.001	0.71 (0.57 to 0.86)	<.001	0.00167
	2009–2018	−0.58 (−1.06 to −0.22)	<.001			
Diseases of the skin and subcutaneous tissue	1998–2013	0.85 (0.62 to 1.19)	<.001	0.2 (0 to 0.42)	.05	0.0714
	2013–2018	−1.73 (−4.29 to −0.64)	.00			
Diseases of musculoskeletal system	1998–2004	−0.22 (−2.91 to 0.77)	.50	0.19 (−0.03 to 0.42)	.09	0.120
	2004–2010	1.92 (0.95 to 4.13)	.03			
	2010–2018	−0.77 (−1.61 to −0.16)	.03			
Diseases of the genitourinary system	1998–2007	−1.1 (−1.81 to −0.59)	.02	0.44 (0.25 to 0.6)	<.001	0.00167
	2007–2011	3.14 (0.26 to 4.94)	.04			
	2011–2018	0.92 (−0.36 to 1.51)	.09			
Pregnancy & childbirth	1998–2007	−0.43 (−0.95 to 0.31)	.15	−3.98 (−4.39 to −3.72)	<.001	0.00167
	2007–2012	−5.13 (−6.43 to −1.99)	.03			
	2012–2016	−10.85 (−13.63 to −7.46)	<.001			
	2016–2018	−2.49 (−8.4 to 1.31)	.13			
Certain conditions	1998–2018	1.02 (0.76 to 1.29)	<.001	1.02 (0.76 to 1.29)	<.001	
Congenital malformations	1998–2014	−0.31 (−0.63 to 0.24)	.19	−1.09 (−1.61 to −0.73)	.01	0.0154
	2014–2018	−4.17 (−9.18 to −1.89)	<.001			
Laboratory findings	1998–2009	0.78 (0.27 to 1.21)	.00	2.88 (2.71 to 3.08)	<.001	0.00167
	2009–2018	5.52 (5.06 to 6.15)	<.001			
Injury & poisoning	1998–2016	0.06 (−0.07 to 1.47)	.23	−0.18 (−0.38 to 0.13)	.10	0.125
	2016–2018	−2.27 (−4.43 to −0.09)	.03			
Factors influencing health status	1998–2013	1.88 (0.87 to 3.37)	.001	−0.62 (−1.75 to 0.29)	.18	0.211
	2013–2018	−7.76 (−17.01 to 3.69)	<.001			

AAPC = average annual percent change, APC = annual percent change, CI = confidence interval.

### 3.2. Trends in types of hospital admission among pediatrics (based on separations)

**Figure 2.**
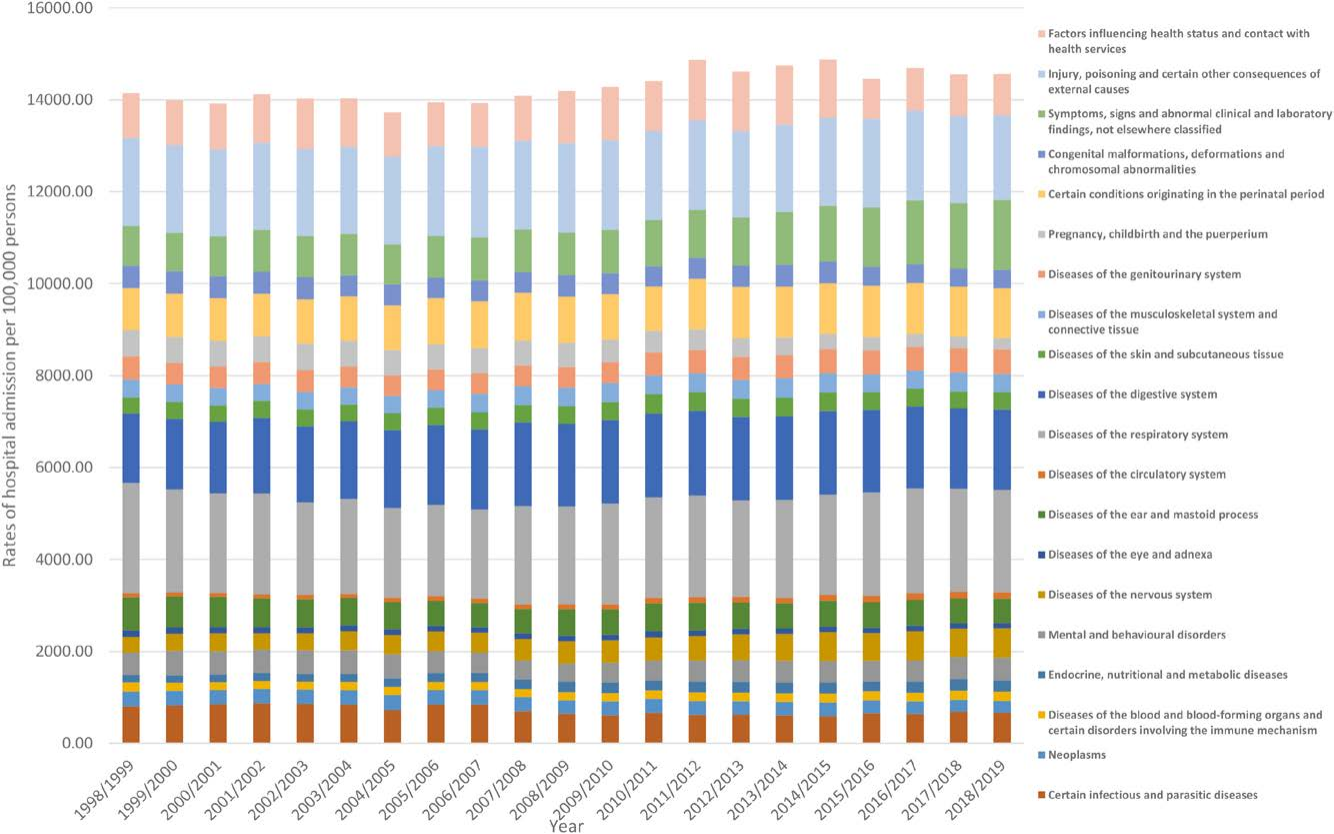
Rates of hospital admission among pediatrics in Australia stratified by type between 1998 and 2019.

During the study time, hospital admission rates among pediatrics for the following: diseases of the nervous system, symptoms, signs and abnormal clinical and laboratory findings, not elsewhere classified, and diseases of the circulatory system increased by 89.7%, 77.7%, and 57.4%, respectively (Table 2). Still, hospital admission rates among pediatrics for the following: pregnancy, childbirth and the puerperium, diseases of the ear and mastoid process, and diseases of the eye and adnexa decreased by 56.0%, 26.1%, and 19.4%, respectively (Table 3, Fig. 2).

**Table 3:** Percentage change in the hospital admission rates among pediatrics from 1998 to 2019 in Australia.

Disorders	Rate of disorders in 1998 per 100,000 persons (95% CI)		Rate of disorders in 2019 per 100,000 persons (95% CI)		Percentage change from 1998–2019
“Certain infectious and parasitic diseases”	805.61 (797.95–813.28)	664.23 (657.85–670.61)		−17.6%	
“Neoplasms”	316.16 (311.35–320.98)	260.93 (256.92–264.93)		−17.5%	
“Diseases of the blood and blood-forming organs and certain disorders involving the immune mechanism”	199.89 (196.06–203.72)	197.74 (194.25–201.23)		−1.1%	
“Endocrine, nutritional and metabolic diseases”	165.48 (161.99–168.97)	253.19 (249.24–257.14)		53.0%	
“Mental and behavioral disorders”	494.18 (488.17–500.20)	488.18 (482.71–493.66)		−1.2%	
“Diseases of the nervous system”	336.99 (332.02–341.96)	639.35 (633.09–645.61)		89.7%	
“Diseases of the eye and adnexa”	144.96 (141.69–148.22)	116.85 (114.17–119.54)		−19.4%	
“Diseases of the ear and mastoid process”	711.68 (704.47–718.89)	525.69 (520.01–531.37)		−26.1%	
“Diseases of the circulatory system”	89.97 (87.40–92.54)	141.63 (138.68–144.59)		57.4%	
“Diseases of the respiratory system”	2404.09 (2390.96–2417.23)	2228.08 (2216.49–2239.67)		−7.3%	
“Diseases of the digestive system”	1502.46 (1492.03–1512.89)	1746.53 (1736.24–1756.82)		16.2%	
“Diseases of the skin and subcutaneous tissue”	358.68 (353.56–363.81)	376.32 (371.51–381.13)		4.9%	
“Diseases of the musculoskeletal system and connective tissue”	380.63 (375.35–385.91)	399.61 (394.65–404.56)		5.0%	
“Diseases of the genitourinary system”	503.18 (497.11–509.25)	528.60 (522.91–534.30)		5.1%	
“Pregnancy, childbirth and the puerperium”	574.62 (568.13–581.10)	253.00 (249.05–256.94)		−56.0%	
“Certain conditions originating in the perinatal period”	917.73 (909.56–925.91)	1082.38 (1074.25–1090.51)		17.9%	
“Congenital malformations, deformations and chromosomal abnormalities”	483.61 (477.66–489.56)	392.27 (387.36–397.18)		−18.9%	
“Symptoms, signs and abnormal clinical and laboratory findings”	861.48 (853.56–869.41)	1530.67 (1521.03–1540.32)		77.7%	
“Injury, poisoning and certain other consequences of external causes”	1926.83 (1915.05–1938.62)	1840.66 (1830.10–1851.22)		−4.5%	
“Factors influencing health status and contact with health services”	971.21 (962.80–979.62)	891.96 (884.58–899.35)		−8.2%	

CI = confidence interval.

### 3.3. Trends in total hospital admission among pediatrics (based on gender)

**Figure 3.**
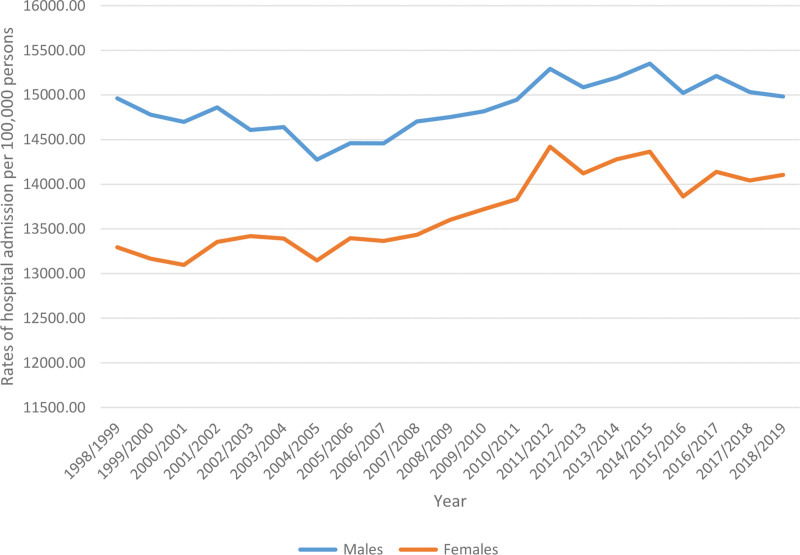
Total hospital admission rates among pediatrics in Australia stratified by gender between 1998 and 2019.

Males accounted for 9,048,416 hospital admission episodes among pediatrics, representing 53.3% of all hospital admission episodes among pediatrics, with a mean of 430,876 cases per year. Hospital admission rates among females rose by 6.1% [from 13,294.5 (95% CI: 13,252.8–13,336.2) in 1998 to 14,105.5 (95% CI: 14,066.2–14,144.7) in 2019 per 100,000 persons]. Hospital admission rates among males rose by 0.1% [from 14,962.3 (95% CI: 14,919.6–15,005.1) in 1998 to 14,982.8 (95% CI: 14,943.8–15,021.9) in 2019 per 100,000 persons] (Fig. 3).

The Joinpoint regression analysis showed similar admission trends for males and females across most diseases. For infectious diseases, both genders experienced sharp declines between 2006 and 2009 (APC = –9.6% for females and –9.57% for males, *P* < .001), with overall decreases (AAPC = –1.23% and –1.24%, respectively, *P* < .001). Neoplasms showed modest long-term declines in both females (AAPC = –1.10%, 95% CI: –1.33 to –0.76, *P* < .001) and males (AAPC = –1.08%, 95% CI: –1.54 to –0.76, *P* < .001), with the steepest drop after 2014 (APC = –3.36% in females and –3.83% in males). Endocrine diseases increased significantly for both genders (AAPC = 2.44% in females; 2.39% in males, *P* < .001), with peaks between 2006 and 2009 in males (APC = 6.94%, *P* = .01) and 2015 and 2018 in females (APC = 5.02%, *P* < .001). Further details are provided in Table 4.

**Table 4:** Trends in hospital admission rates by disease category and gender: Joinpoint regression analysis, 1998 to 2018.

Disease	Gender	Range	APC (95% CI)	*P*-value	AAPC (95% CI)	*P*-value
Infections	Female	1998–2006	−0.44 (−1.53 to 2.17)	.70	−1.23 (−1.67 to −0.65)	.00
	Female	2006–2009	−9.6 (−12.36 to −3.9)	.00		
	Female	2009–2018	1 (−0.25 to 3.82)	.10		
	Male	1998–2006	−0.45 (−1.55 to 2.18)	.70	−1.24 (−1.67 to −0.65)	.00
	Male	2006–2009	−9.57 (−12.32 to −3.86)	.00		
	Male	2009–2018	1 (−0.27 to 3.9)	.10		
Neoplasm	Female	1998–2014	−0.52 (−0.77 to 0.05)	.06	−1.1 (−1.33 to −0.76)	<.001
	Female	2014–2018	−3.36 (−6.69 to −1.39)	.00		
	Male	1998–2014	−0.38 (−0.68 to 0.11)	.09	−1.08 (−1.54 to −0.76)	<.001
	Male	2014–2018	−3.83 (−8.28 to −1.79)	<.001		
Blood	Female	1998–2000	−5.72 (−8.17 to −0.29)	.04	1.36 (1.13 to 1.79)	<.001
	Female	2000–2009	1.03 (−0.06 to 2.58)	.06		
	Female	2009–2018	3.34 (2.67 to 5.24)	.01		
	Male	1998–2018	−0.54 (−0.87 to −0.21)	.00	−0.54 (−0.87 to −0.21)	.00
Endocrine	Female	1998–2009	2.95 (2.41 to 3.88)	.01	2.44 (2.13 to 2.74)	<.001
	Female	2009–2015	0.27 (−2.79 to 1.67)	.92		
	Female	2015–2018	5.02 (1.96 to 9.05)	<.001		
	Male	1998–2006	3.04 (−0.14 to 4.84)	.06	2.39 (2.06 to 2.69)	<.001
	Male	2006–2009	6.94 (2.04 to 8.75)	.01		
	Male	2009–2015	−0.98 (−3.7 to 4.99)	.08		
	Male	2015–2018	3.06 (0.25 to 6.7)	.03		
Mental	Female	1998–2004	3.17 (0.93 to 9.7)	.03	1.44 (0.91 to 2.27)	.01
	Female	2004–2007	−7.89 (−10.91 to −0.78)	.03		
	Female	2007–2018	3.18 (2 to 5.83)	.02		
	Male	1998–2008	−4.01 (−6.54 to −3.11)	.00	−2.53 (−2.99 to −2.09)	<.001
	Male	2008–2018	−1.03 (−2.07 to 1.92)	.26		
Nervous	Female	1998–2016	3.87 (3.27 to 8.05)	.01	3.42 (3.03 to 4.2)	<.001
	Female	2016–2018	−0.55 (−4.76 to 3.79)	.90		
	Male	1998–2014	3.72 (3.27 to 12.95)	.00	3.18 (2.59 to 4.27)	<.001
	Male	2014–2018	1.04 (−5.44 to 3.38)	.46		
Eye	Female	1998–2006	−2.09 (−3.45 to −1.52)	.00	−1.21 (−1.44 to −1.03)	<.001
	Female	2006–2010	1.29 (−0.31 to 3.12)	.12		
	Female	2010–2018	−1.55 (−2.59 to −1.1)	.00		
	Male	1998–2005	−2.14 (−3.11 to −1.32)	.02	−0.98 (−1.19 to −0.84)	<.001
	Male	2005–2011	0.4 (−2.64 to 2.06)	.30		
	Male	2011–2016	−1.88 (−3.48 to 0.84)	.13		
	Male	2016–2018	1.26 (−1.36 to 3.05)	.33		
Ear	Female	1998–2006	−3.68 (−4.62 to −3.03)	<.001	−1.39 (−1.63 to −1.2)	<.001
	Female	2006–2010	2.92 (0.94 to 5.41)	.00		
	Female	2010–2018	−1.19 (−2.26 to −0.64)	.00		
	Male	1998–2006	−3.46 (−5.04 to −2.58)	.01	−1.39 (−1.74 to −1.15)	<.001
	Male	2006–2010	2.54 (0.16 to 5.4)	.04		
	Male	2010–2018	−1.23 (−3 to −0.55)	.01		
Cicuralorty	Female	1998–2003	0.86 (−3.84 to 3.11)	.56	2.77 (2.43 to 3.29)	<.001
	Female	2003–2018	3.41 (2.86 to 5.56)	.03		
	Male	1998–2018	2.74 (2.32 to 3.21)	<.001	2.74 (2.32 to 3.21)	<.001
Respiratory	Female	1998–2002	−3.93 (−9.36 to −0.75)	.01	−0.2 (−0.53 to 0.32)	.34
	Female	2002–2018	0.76 (0.35 to 1.71)	.01		
	Male	1998–2004	−2.69 (−9.17 to −0.31)	.02	−0.13 (−0.56 to 0.46)	.55
	Male	2004–2018	0.98 (0.33 to 3.6)	.02		
Digestive	Female	1998–2007	2.24 (1.93 to 2.82)	<.001	0.67 (0.53 to 0.82)	<.001
	Female	2007–2013	0.43 (−0.32 to 1.24)	.20		
	Female	2013–2018	−1.8 (−3.01 to −1.18)	.00		
	Male	1998–2007	1.73 (1.45 to 2.12)	<.001	0.75 (0.65 to 0.86)	<.001
	Male	2007–2018	−0.05 (−0.27 to 0.16)	.63		
Skin	Female	1998–2003	−0.65 (−3.29 to 0.47)	.25	0.37 (0.13 to 0.59)	.00
	Female	2003–2013	1.72 (1.33 to 3.5)	.00		
	Female	2013–2018	−1.26 (−3 to −0.28)	.02		
	Male	1998–2010	0.99 (0.65 to 1.46)	<.001	−0.01 (−0.22 to 0.2)	1.00
	Male	2010–2018	−1.47 (−2.31 to −0.9)	<.001		
Muscoskeletal	Female	1998–2003	−1.28 (−4.21 to 0.27)	.08	0.69 (0.4 to 0.93)	<.001
	Female	2003–2013	2.13 (0.55 to 3.75)	.04		
	Female	2013–2016	−2.27 (−3.7 to 3.24)	.18		
	Female	2016–2018	3.02 (−0.8 to 5.67)	.15		
	Male	1998–2010	0.86 (0.45 to 1.5)	<.001	−0.07 (−0.36 to 0.19)	.59
	Male	2010–2018	−1.44 (−2.63 to −0.76)	<.001		
Gent	Female	1998–2006	−1.16 (−2.29 to −0.4)	.01	0.62 (0.38 to 0.85)	<.001
	Female	2006–2018	1.82 (1.42 to 2.39)	<.001		
	Male	1998–2004	−2.04 (−3.77 to −1.04)	.01	0.13 (−0.12 to 0.35)	.21
	Male	2004–2015	1.61 (1.26 to 3.47)	.02		
	Male	2015–2018	−0.88 (−3.7 to 0.93)	.33		
Pregnancy	Female	1998–2007	−0.4 (−0.91 to 0.3)	.17	−3.95 (−4.36 to −3.7)	<.001
	Female	2007–2012	−5.13 (−6.41 to −2.41)	.02		
	Female	2012–2016	−10.85 (−13.6 to −8.69)	<.001		
	Female	2016–2018	−2.38 (−8.13 to 1.36)	.14		
Certain condition	Female	1998–2018	0.98 (0.73 to 1.24)	<.001	0.98 (0.73 to 1.24)	<.001
	Male	1998–2018	1.06 (0.78 to 1.34)	<.001	1.06 (0.78 to 1.34)	<.001
Conginetal	Female	1998–2014	−0.41 (−0.75 to 0.04)	.07	−1.51 (−2.02 to −1.18)	<.001
	Female	2014–2018	−5.78 (−10.48 to −3.43)	<.001		
	Male	1998–2005	−1.4 (−3.36 to −0.63)	.00	−1.09 (−1.36 to −0.87)	<.001
	Male	2005–2014	0.49 (−0.03 to 2.53)	.07		
	Male	2014–2018	−4.05 (−6.4 to −2.48)	<.001		
Lab	Female	1998–2009	1.27 (0.73 to 1.72)	.00	2.93 (2.75 to 3.13)	<.001
	Female	2009–2018	5 (4.52 to 5.66)	<.001		
	Male	1998–2009	0.26 (−0.33 to 0.76)	.32	2.84 (2.64 to 3.06)	<.001
	Male	2009–2018	6.08 (5.54 to 6.81)	<.001		
Injury	Female	1998–2009	0.11 (−0.64 to 0.49)	.60	0.44 (0.23 to 0.64)	<.001
	Female	2009–2016	1.64 (0.65 to 3.5)	.04		
	Female	2016–2018	−1.9 (−4.27 to 0.91)	.15		
	Male	1998–2009	0.32 (0.16 to 0.54)	.01	−0.5 (−0.61 to −0.39)	<.001
	Male	2009–2012	−2.48 (−3.08 to −1.39)	.02		
	Male	2012–2016	0.1 (−0.42 to 1.09)	.47		
	Male	2016–2018	−3.18 (−4.47 to −1.7)	<.001		
Health status	Female	1998–2013	3.35 (2.24 to 4.87)	<.001	0.19 (−0.93 to 1.16)	.65
	Female	2013–2018	−8.7 (−16.23 to −4.44)	<.001		
	Male	1998–2006	−1.74 (−7.64 to 0.13)	.07	−1.63 (−2.44 to −1.03)	<.001
	Male	2006–2012	4.44 (1.54 to 11.63)	.01		
	Male	2012–2018	−7.22 (−11.01 to −4.77)	.00		

AAPC = average annual percent change, APC = annual percent change, CI = confidence interval.

### 3.4. Trends in total hospital admission among pediatrics (based on age group)

**Figure 4.**
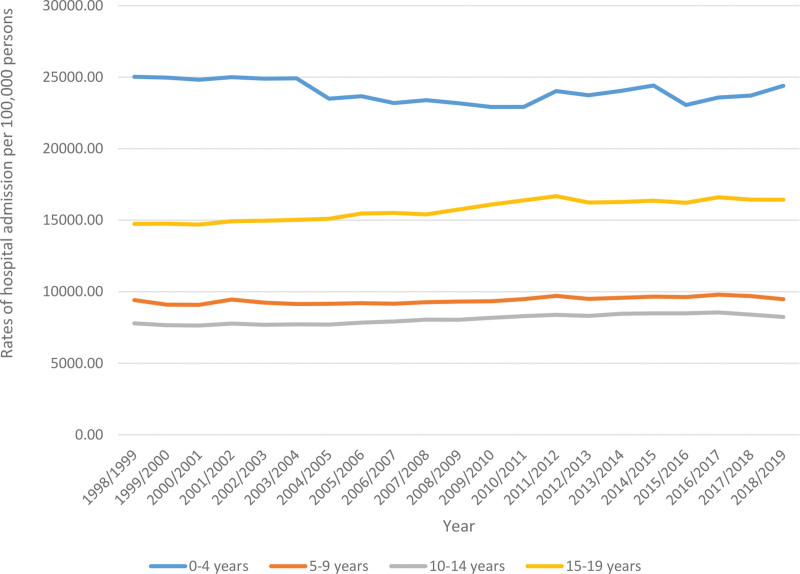
Total hospital admission rates among pediatrics in Australia stratified by age group between 1998 and 2019.

Concerning age group differences in hospital admission among pediatrics, the age group 0 to 4 years accounted for 41.8% of the total number of hospital admission among pediatrics, followed by the age group 15 to 19 years with 27.7%, the age group 5 to 9 years with 16.5%, and then 10 to 14 years with 14.0%. Rates of hospital admission among pediatrics aged 0 to 4 years decreased by 2.5% [from 25,026.3 (95% CI: 24,951.2–25,101.3) in 1998 to 24,394.5 (95% CI: 24,326.9–24,462.0) in 2019 per 100,000 persons]. Rates of hospital admission among pediatrics aged 5 to 9 years increased by 0.6% [from 9417.7 (95% CI: 9368.2–9467.3) in 1998 to 9473.1 (95% CI: 9428.0–9518.3) in 2019 per 100,000 persons]. Rates of hospital admission among pediatrics aged 10 to 14 years increased by 5.7% [from 7789.3 (95% CI: 7743.5–7835.1) in 1998 to 8235.1 (95% CI: 8192.–8278.2) in 2019 per 100,000 persons]. Rates of hospital admission among pediatrics aged 15 to 19 years increased by 11.5% [from 14,741.1 (95% CI: 14,680.0–14,802.2) in 1998 to 16,430.1 (95% CI: 16,370.7–16,489.5) in 2019 per 100,000 persons] (Fig. 4).

The Joinpoint regression revealed heterogeneous age-specific patterns in hospital admissions. For infectious diseases, the sharpest fall was observed in children aged 0 to 4 years between 2006 and 2009 (APC = –15.81%, 95% CI: –19.24 to –8.32, *P* < .001), while adolescents aged 15 to 19 showed a temporary increase (APC = 10.8%, 2006–2009, *P* < .001). Neoplasms declined across all ages, with the steepest reductions in 10 to 14 years between 2015 and 2018 (APC = –7.4%, *P* < .001) and in 5 to 9 years from 1998 to 2000 (APC = –7.43%, *P* < .001). Endocrine diseases rose significantly in adolescents 15 to 19 years, especially between 2015 and 2018 (APC = 8.66%, *P* < .001), while younger groups showed mixed trends (Table 5).

**Table 5:** Trends in hospital admission rates by disease category and age group: Joinpoint regression analysis, 1998 to 2018.

Disease	Cohort	Range	APC (95% CI)	*P*-value	AAPC (95% CI)	*P*-value
Certain infections	0–4 yr	1998–2006	−0.59 (−2.06 to 2.23)	.67	−1.7 (−2.28 to −1.02)	<.001
	0–4 yr	2006–2009	−15.81 (−19.24 to −8.32)	<.001		
	0–4 yr	2009–2018	2.47 (0.75 to 5.51)	<.001		
	5–9 yr	1998–2018	−2.34 (−3.42 to −1.36)	<.001	−2.34 (−3.42 to −1.36)	<.001
	10–14 yr	1998–2006	0.43 (−0.94 to 6.08)	.49	−1.26 (−1.94 to −0.56)	<.001
	10–14 yr	2006–2018	−2.36 (−5.1 to −1.62)	<.001		
	15–19 yr	1998–2006	−0.66 (−4.72 to 0.9)	.31	1.33 (0.78 to 1.81)	<.001
	15–19 yr	2006–2009	10.8 (4.51 to 14.23)	<.001		
	15–19 yr	2009–2018	0.1 (−1.56 to 1.02)	.97		
Neoplasm	0–4 yr	1998–2018	−0.71 (−1.29 to −0.11)	.02	−0.71 (−1.29 to −0.11)	.02
	5–9 yr	1998–2000	−7.43 (−11.09 to −1.33)	<.001	−1.42 (−1.86 to −0.92)	<.001
	5–9 yr	2000–2012	−0.04 (−0.49 to 4.17)	.63		
	5–9 yr	2012–2018	−2.11 (−6.39 to −0.83)	.01		
	10–14 yr	1998–2000	5.75 (0.59 to 11.25)	.02	−0.91 (−1.37 to −0.49)	<.001
	10–14 yr	2000–2012	−1.01 (−3.96 to −0.74)	<.001		
	10–14 yr	2012–2015	1.93 (−0.49 to 3.7)	.22		
	10–14 yr	2015–2018	−7.4 (−11.39 to −4.91)	<.001		
	15–19 yr	1998–2004	0.64 (−0.52 to 3.96)	.27	−0.9 (−1.21 to −0.54)	<.001
	15–19 yr	2004–2018	−1.55 (−2.23 to −1.2)	<.001		
Blood	0–4 yr	1998–2000	−10.67 (−14.92 to −0.34)	.03	−0.7 (−1.16 to 0.28)	.14
	0–4 yr	2000–2018	0.48 (−0.09 to 2.21)	.08		
	5–9 yr	1998–2018	−0.49 (−1.25 to 0.28)	.20	−0.49 (−1.25 to 0.28)	.20
	10–14 yr	1998–2018	−0.05 (−0.56 to 0.46)	.87	−0.05 (−0.56 to 0.46)	.87
	15–19 yr	1998–2000	−5.49 (−8.4 to 1.19)	.11	2.05 (1.7 to 2.62)	<.001
	15–19 yr	2000–2014	2.14 (1.16 to 3)	.01		
	15–19 yr	2014–2018	5.76 (3.47 to 10.18)	<.001		
Endocrine	0–4 yr	1998–2015	0.73 (−1.35 to 1.31)	.17	1.53 (0.86 to 1.96)	<.001
	0–4 yr	2015–2018	6.17 (1.34 to 12.26)	<.001		
	5–9 yr	1998–2009	4.28 (3.4 to 5.73)	<.001	2.19 (1.75 to 2.7)	<.001
	5–9 yr	2009–2018	−0.31 (−1.76 to 0.74)	.52		
	10–14 yr	1998–2010	3.95 (3.35 to 4.75)	<.001	1.55 (1.19 to 1.9)	<.001
	10–14 yr	2010–2018	−1.94 (−3.26 to −0.98)	<.001		
	15–19 yr	1998–2003	1.95 (−1.86 to 3.4)	.17	3.72 (3.42 to 4.01)	<.001
	15–19 yr	2003–2008	5.95 (4.53 to 8.85)	<.001		
	15–19 yr	2008–2015	1.37 (−0.59 to 2.11)	.10		
	15–19 yr	2015–2018	8.66 (6.11 to 12.87)	<.001		
Mental	0–4 yr	1998–2008	−13.72 (−18.09 to −11.47)	.01	−9.51 (−11.62 to −8.42)	<.001
	0–4 yr	2008–2011	14.42 (−4.18 to 24.98)	.18		
	0–4 yr	2011–2018	−12.41 (−26.89 to −7.73)	<.001		
	5–9 yr	1998–2005	0.67 (−3.92 to 5.59)	.42	−6.59 (−7.74 to −5.73)	<.001
	5–9 yr	2005–2008	−13.54 (−18.52 to 2.93)	.06		
	5–9 yr	2008–2011	3.08 (−18.11 to 9.61)	.58		
	5–9 yr	2011–2018	−14.11 (−20.2 to −6.49)	.02		
	10–14 yr	1998–2018	−1.51 (−2.1 to −0.91)	<.001	−1.51 (−2.1 to −0.91)	<.001
	15–19 yr	1998–2004	1.36 (0.23 to 4.22)	.03	1.33 (1.04 to 1.79)	<.001
	15–19 yr	2004–2007	−7.52 (−9.54 to −3.16)	.01		
	15–19 yr	2007–2018	3.88 (3.19 to 4.87)	<.001		
Disease of nervous system	0–4 yr	1998–2018	1.18 (0.57 to 1.84)	<.001	1.18 (0.57 to 1.84)	<.001
	5–9 yr	1998–2005	5.98 (2.21 to 7.2)	.01	5.3 (4.96 to 5.62)	<.001
	5–9 yr	2005–2008	10.57 (7.51 to 12.33)	<.001		
	5–9 yr	2008–2016	5.16 (4.35 to 5.93)	<.001		
	5–9 yr	2016–2018	−3.83 (−6.25 to −0.42)	.03		
	10–14 yr	1998–2013	5.29 (4.96 to 6.5)	<.001	4.72 (4.34 to 5.16)	<.001
	10–14 yr	2013–2018	3.01 (−0.65 to 4.43)	.06		
	15–19 yr	1998–2001	−1.74 (−7.35 to 3.65)	.46	4.32 (3.99 to 4.96)	<.001
	15–19 yr	2001–2018	5.43 (5.11 to 6.06)	<.001		
Disease of eye amd adnexa	0–4 yr	1998–2006	−3.74 (−6.81 to −2.08)	.04	−2.85 (−3.3 to −2.44)	<.001
	0–4 yr	2006–2009	−0.12 (−4.55 to 1.61)	.57		
	0–4 yr	2009–2018	−2.96 (−7.22 to −2.1)	.02		
	5–9 yr	1998–2018	−0.29 (−0.5 to −0.08)	.01	−0.29 (−0.5 to −0.08)	.01
	10–14 yr	1998–2018	0.58 (0.16 to 1)	.01	0.58 (0.16 to 1)	.01
	15–19 yr	1998–2008	−0.1 (−3.76 to 3.09)	.66	1.14 (0.5 to 1.59)	<.001
	15–19 yr	2008–2011	7.22 (−3.35 to 10.19)	.10		
	15–19 yr	2011–2016	−2.55 (−8.19 to 6.64)	.13		
	15–19 yr	2016–2018	8.12 (−0.87 to 14.74)	.10		
Disease of ear and mastoid	0–4 yr	1998–2006	−3.07 (−6.57 to −1.71)	<.001	−1.08 (−1.54 to −0.6)	<.001
	0–4 yr	2006–2018	0.27 (−0.48 to 2.01)	.44		
	5–9 yr	1998–2004	−3.45 (−7.26 to −2.07)	.01	−2.13 (−2.47 to −1.75)	<.001
	5–9 yr	2004–2018	−1.56 (−2.06 to 0.63)	.07		
	10–14 yr	1998–2000	−5.54 (−7.89 to −1.1)	<.001	−1.62 (−1.95 to −1.2)	<.001
	10–14 yr	2000–2016	−0.49 (−0.7 to 1.38)	.23		
	10–14 yr	2016–2018	−6.43 (−9.86 to −1.62)	<.001		
	15–19 yr	1998–2018	0.23 (−0.21 to 0.68)	.27	0.23 (−0.21 to 0.68)	.27
Circulatory system	0–4 yr	1998–2002	−2.66 (−10.08 to 1.76)	.26	1.92 (1.43 to 2.65)	<.001
	0–4 yr	2002–2018	3.1 (2.59 to 4.62)	.01		
	5–9 yr	1998–2004	0.51 (−7.43 to 3.67)	.86	3.98 (3.43 to 4.77)	<.001
	5–9 yr	2004–2018	5.51 (4.82 to 7.58)	.00		
	10–14 yr	1998–2002	−0.96 (−7.2 to 2.33)	.58	2.31 (1.84 to 2.89)	<.001
	10–14 yr	2002–2018	3.14 (2.74 to 4.49)	.01		
	15–19 yr	1998–2007	2.02 (−0.94 to 5.12)	.09	2.36 (2 to 2.77)	<.001
	15–19 yr	2007–2010	7.92 (−0.52 to 10.05)	.06		
	15–19 yr	2010–2013	−2.41 (−4.56 to 7.06)	.20		
	15–19 yr	2013–2018	2.67 (0.63 to 7.31)	.02		
Respiratory system	0–4 yr	1998–2005	−2.35 (−4.48 to −1.24)	<.001	−0.27 (−0.56 to 0.03)	.07
	0–4 yr	2005–2018	0.86 (0.42 to 1.53)	<.001		
	5–9 yr	1998–2002	−4.27 (−9.94 to −0.59)	.01	−0.77 (−1.18 to −0.11)	.02
	5–9 yr	2002–2018	0.13 (−0.45 to 2.43)	.45		
	10–14 yr	1998–2004	−5.02 (−10.22 to −2.96)	.01	−0.99 (−1.57 to −0.55)	<.001
	10–14 yr	2004–2009	3.52 (0.84 to 9.04)	.02		
	10–14 yr	2009–2018	−0.69 (−3.71 to 0.23)	.12		
	15–19 yr	1998–2004	−1.96 (−5.79 to −0.39)	.02	0.34 (−0.03 to 0.67)	.08
	15–19 yr	2004–2009	4.16 (2.08 to 8.06)	<.001		
	15–19 yr	2009–2018	−0.19 (−1.47 to 0.49)	.53		
Digestive system	0–4 yr	1998–2005	−1.53 (−2.08 to −0.52)	.03	−2.09 (−2.28 to −1.89)	<.001
	0–4 yr	2005–2013	−3.58 (−5.57 to −3.08)	<.001		
	0–4 yr	2013–2018	−0.45 (−1.61 to 2.19)	.45		
	5–9 yr	1998–2003	5.2 (4.13 to 7.39)	<.001	1.97 (1.79 to 2.17)	<.001
	5–9 yr	2003–2011	2.09 (1.35 to 2.79)	<.001		
	5–9 yr	2011–2018	−0.41 (−1.18 to 0.08)	.10		
	10–14 yr	1998–2013	2.16 (1.96 to 2.4)	<.001	1.46 (1.28 to 1.62)	<.001
	10–14 yr	2013–2018	−0.61 (−1.92 to 0.22)	.13		
	15–19 yr	1998–2009	2.67 (2.44 to 3.1)	<.001	1.27 (1.12 to 1.42)	<.001
	15–19 yr	2009–2014	0.62 (−0.19 to 2.1)	.11		
	15–19 yr	2014–2018	−1.72 (−3.27 to −0.85)	<.001		
Skin and subcutanous tissue	0–4 yr	1998–2018	0.37 (−0.01 to 0.78)	.05	0.37 (−0.01 to 0.78)	.05
	5–9 yr	1998–2013	0.8 (0.52 to 1.76)	<.001	0.29 (−0.06 to 0.64)	.08
	5–9 yr	2013–2018	−1.24 (−4.76 to 0.11)	.07		
	10–14 yr	1998–2018	−0.61 (−0.81 to −0.39)	<.001	−0.61 (−0.81 to −0.39)	<.001
	15–19 yr	1998–2011	1.59 (1.31 to 1.99)	<.001	0.92 (0.73 to 1.11)	<.001
	15–19 yr	2011–2018	−0.3 (−1.29 to 0.3)	.34		
Muscoskeletal	0–4 yr	1998–2018	0.65 (0.37 to 0.95)	<.001	0.65 (0.37 to 0.95)	<.001
	5–9 yr	1998–2008	2 (0.87 to 6.46)	<.001	0.44 (−0.2 to 1.15)	.15
	5–9 yr	2008–2018	−1.1 (−4.57 to 0)	.05		
	10–14 yr	1998–2003	−0.41 (−3.62 to 0.99)	.55	0.69 (0.38 to 0.98)	.00
	10–14 yr	2003–2013	1.96 (1.5 to 4.09)	.02		
	10–14 yr	2013–2016	−3.81 (−5.56 to −0.66)	.05		
	10–14 yr	2016–2018	4.16 (−0.27 to 7.32)	.06		
	15–19 yr	1998–2004	−1.63 (−2.47 to −1.06)	.01	−0.04 (−0.18 to 0.09)	.48
	15–19 yr	2004–2010	2.26 (−0.63 to 3.49)	.07		
	15–19 yr	2010–2015	0.27 (−0.32 to 2.16)	.30		
	15–19 yr	2015–2018	−1.89 (−3.58 to −0.7)	<.001		
Genitourinary system	0–4 yr	1998–2008	−1.97 (−3.32 to 0)	.05	−0.75 (−1.1 to −0.44)	<.001
	0–4 yr	2008–2011	3.22 (−3.18 to 4.93)	.12		
	0–4 yr	2011–2018	−0.66 (−3.59 to 0.44)	.15		
	5–9 yr	1998–2003	−1.98 (−5.78 to −0.3)	.02	0.13 (−0.12 to 0.46)	.32
	5–9 yr	2003–2018	0.84 (0.52 to 1.61)	<.001		
	10–14 yr	1998–2000	−6.27 (−8.38 to −1.68)	.01	0.98 (0.71 to 1.35)	<.001
	10–14 yr	2000–2008	1.56 (−0.16 to 2.54)	.06		
	10–14 yr	2008–2015	3.38 (2.75 to 5.65)	<.001		
	10–14 yr	2015–2018	−1.05 (−4.01 to 0.8)	.23		
	15–19 yr	1998–2007	−1.1 (−1.73 to −0.55)	<.001	1.18 (1 to 1.36)	<.001
	15–19 yr	2007–2018	3.08 (2.73 to 3.53)	<.001		
Pregnancy & childbirth	10–14 yr	1998–2002	6.58 (3.7 to 14.32)	<.001	−3.99 (−4.67 to −3.32)	<.001
	10–14 yr	2002–2005	−7.61 (−11.46 to −1.82)	.01		
	10–14 yr	2005–2008	7.03 (−7.57 to 11.31)	.08		
	10–14 yr	2008–2018	−9.83 (−11.69 to −8.66)	<.001		
	15–19 yr	1998–2007	−0.99 (−1.64 to −0.09)	.04	−3.79 (−4.23 to −3.54)	<.001
	15–19 yr	2007–2012	−4.33 (−5.81 to −1.23)	.03		
	15–19 yr	2012–2016	−9.98 (−12.83 to −4.8)	<.001		
	15–19 yr	2016–2018	−2.08 (−8.05 to 1.77)	.18		
Certain conditions	0–4 yr	1998–2006	1.41 (0.88 to 2.33)	.02	0.68 (0.47 to 0.89)	<.001
	0–4 yr	2006–2009	−3.28 (−4.7 to −0.43)	.05		
	0–4 yr	2009–2012	3.76 (1.49 to 5.3)	.04		
	0–4 yr	2012–2018	0.22 (−1.24 to 0.91)	.68		
	5–9 yr	1998–2018	−6.9 (−9.86 to −4.71)	<.001	−6.9 (−9.86 to −4.71)	<.001
	10–14 yr	1998–2001	71.58 (28.03 to 222.5)	<.001	−6.39 (−10.84 to −2.19)	.01
	10–14 yr	2001–2018	−15.88 (−20.6 to −13.75)	<.001		
	15–19 yr	1998–2001	−23.05 (−55.89 to 13.33)	.18	−3.83 (−8.3 to 1.02)	.11
	15–19 yr	2001–2007	42.93 (29.48 to 83.22)	.03		
	15–19 yr	2007–2010	−52.46 (−63.78 to −25.83)	.03		
	15–19 yr	2010–2018	1.17 (−13.44 to 32.77)	.70		
Congenital malformation	0–4 yr	1998–2014	−0.57 (−0.87 to −0.16)	.01	−1.48 (−1.84 to −1.17)	<.001
	0–4 yr	2014–2018	−5.05 (−8.75 to −2.91)	<.001		
	5–9 yr	1998–2002	1.05 (−0.7 to 4.98)	.17	−1.15 (−1.4 to −0.79)	<.001
	5–9 yr	2002–2005	−4.53 (−5.87 to −1.81)	.02		
	5–9 yr	2005–2018	−1.02 (−1.44 to 0.35)	.08		
	10–14 yr	1998–2010	−1.22 (−1.8 to −0.01)	.05	−0.85 (−1.1 to −0.68)	<.001
	10–14 yr	2010–2013	2.47 (−2.62 to 3.52)	.23		
	10–14 yr	2013–2016	−3.88 (−5.29 to 2.35)	.22		
	10–14 yr	2016–2018	1.16 (−2.52 to 3.57)	.41		
	15–19 yr	1998–2018	0.07 (−0.3 to 0.44)	.68	0.07 (−0.3 to 0.44)	.68
Laboratory findings	0–4 yr	1998–2011	−0.63 (−1.37 to 0)	.05	2.43 (2.1 to 2.78)	<.001
	0–4 yr	2011–2018	8.36 (7.03 to 10.28)	<.001		
	5–9 yr	1998–2009	0.76 (−0.03 to 1.36)	.06	2.78 (2.55 to 3.05)	<.001
	5–9 yr	2009–2018	5.3 (4.66 to 6.23)	<.001		
	10–14 yr	1998–2008	1.42 (−1.51 to 2.14)	.14	2.26 (1.94 to 2.59)	<.001
	10–14 yr	2008–2018	3.1 (2.49 to 5.56)	<.001		
	15–19 yr	1998–2004	1.64 (−4.12 to 3.86)	.29	4.07 (3.71 to 4.62)	<.001
	15 to 19 yr	2004–2018	5.14 (4.67 to 6.64)	<.001		
Injury	0–4 yr	1998–2007	−1.02 (−1.86 to −0.58)	<.001	−0.24 (−0.4 to −0.07)	.01
	0–4 yr	2007–2018	0.41 (0.11 to 0.97)	.01		
	5–9 yr	1998–2010	−0.7 (−1.41 to −0.49)	.01	−0.69 (−0.87 to −0.56)	<.001
	5–9 yr	2010–2016	0.26 (−0.25 to 1.53)	.28		
	5–9 yr	2016–2018	−3.47 (−5.34 to −1.38)	<.001		
	10–14 yr	1998–2006	0.57 (0.27 to 1.01)	.02	−0.33 (−0.44 to −0.22)	<.001
	10–14 yr	2006–2012	−0.67 (−1.67 to −0.26)	.03		
	10–14 yr	2012–2016	0.66 (0.04 to 1.55)	.04		
	10–14 yr	2016–2018	−4.77 (−6.05 to −3.09)	<.001		
	15–19 yr	1998–2002	−0.48 (−3.44 to 1.36)	.42	0.38 (0.16 to 0.63)	<.001
	15–19 yr	2002–2006	2.92 (−0.55 to 4.82)	.06		
	15–19 yr	2006–2018	−0.16 (−0.64 to 0.34)	.28		
Health status	0–4 yr	1998–2013	2.62 (0.89 to 5.34)	<.001	−1.9 (−4.36 to −0.21)	.03
	0–4 yr	2013–2018	−14.31 (−30.24 to −7.37)	<.001		
	5–9 yr	1998–2007	−0.09 (−5.21 to 5.73)	.76	0.98 (0.44 to 1.67)	<.001
	5–9 yr	2007–2018	1.86 (−2.86 to 6.82)	.06		
	10–14 yr	1998–2018	1.4 (0.98 to 1.86)	<.001	1.4 (0.98 to 1.86)	<.001
	15–19 yr	1998–2013	0.07 (−0.4 to 0.79)	.68	−0.93 (−1.46 to −0.5)	.01
	15–19 yr	2013–2018	−3.87 (−8.91 to −1.84)	<.001		

AAPC = average annual percent change, APC = annual percent change, CI = confidence interval.

### 3.5. Trends in types of hospital admission rates among pediatrics by gender

Most hospital admission rates among pediatrics were higher among males compared to females (Figure S3 A–C, Supplemental Digital Content, https://links.lww.com/MD/R523). Still, hospital admission rates among pediatrics for neoplasms, endocrine, nutritional and metabolic diseases, mental and behavioral disorders, diseases of the eye and adnexa, diseases of the digestive system, pregnancy, childbirth and the puerperium, and symptoms, signs and abnormal clinical and laboratory findings were higher among females compared to males (Figure S3 A–C, Supplemental Digital Content, https://links.lww.com/MD/R523).

### 3.6. Trends in types of hospital admission rates among pediatrics by age

Most types of hospital admission rates for pediatrics were more common among the age group from 0 to 4 years, which include the following: certain infectious and parasitic diseases, neoplasms, diseases of the blood and blood-forming organs and certain disorders involving the immune mechanism, diseases of the nervous system, diseases of the eye and adnexa, diseases of the ear and mastoid process, diseases of the respiratory system, certain conditions originating in the perinatal period, congenital malformations, deformations and chromosomal abnormalities, symptoms, signs and abnormal clinical and laboratory findings, not elsewhere classified, and factors influencing health status and contact with health services. Still, hospital admission rates among pediatrics were more common among the age group from 15 to 19 years for the following: endocrine, nutritional and metabolic diseases, mental and behavioral disorders, diseases of the circulatory system, diseases of the digestive system, diseases of the skin and subcutaneous tissue, diseases of the musculoskeletal system and connective tissue, diseases of the genitourinary system, pregnancy, childbirth and the puerperium, and injury, poisoning and certain other consequences of external causes (Figure S4 A–C, Supplemental Digital Content, https://links.lww.com/MD/R523).

## 4. Discussion

Data presented here were obtained from a longitudinal, consistently maintained, health surveillance system in Australia, facilitating comparison by age and gender over time. Such a comparison is an essential component of understanding and improving the quality of care. This study provides the 1st description of the changes in hospital admissions and admission rates for spectrum of illnesses among pediatric patients between 1998 and 2019.

The mortality rate in Australia decreased by 42% from 6.45 deaths in 1998 to 3.74 per 1000 live births in 2018 among children under 5 years, and there was a decline in the probability of dying among children aged 5 to 9 years from 180 deaths in 1998 to 117 per 1000 children in 2018, and from 202 deaths to 137 per 1000 children aged 10 to 14 years.^[18]^ However, the hospital admission rate for various reasons in our study showed a slight increase of 2.9% within the same period. The improved systems used in acute pediatric healthcare settings in Australia that help in identifying early signs of clinical deterioration and facilitating timely intervention^[19]^ could be one of the reasons why in hospital mortality rate among pediatric patients is reduced. The rise in pediatric admissions was also observed in the UK^[8]^ and Canada,^[20]^ whereas the USA demonstrated a 21% reduction in annual pediatric admissions from 2010 to 2016.^[21]^ These contrasting trends may partly reflect differences in health system organization. Australia, the UK, and Canada operate publicly funded health care models that may facilitate hospital-based care, while USA’s mixed funding model and greater reliance on outpatient services may contribute to declining admission rates.^[22,23]^

A pediatric patient receives hospital-based services either in the same-day or through overnight admission. There are several possible explanations for the divergence of the services received in the same day (rising) and overnight admission rate (falling) among pediatrics over the 20 years. Firstly, a relatively high proportion of children (10–30%) visiting an ED were discharged with no treatment or presented with conditions that could be managed in a primary care setting.^[24]^ Sills et al also noted that unnecessary hospital admissions were common in up to 30% of hospitalized children^[25]^ and an estimated 10% of infants under 1 year attending the ED had no underlying medical problem.^[26]^ Pediatricians, through working with local GPs and social care partners in health information campaigns, can reduce unnecessary hospital trips. Secondly, the overnight-stay hospital admission of a child may carry a risk of iatrogenic complications including nosocomial infections, medication errors, psychological stress, and readmission.^[27]^ Zhou et al reported that children at a tertiary children’s hospital in Western Australia with a longer hospital stay were at a higher risk of being readmitted.^[28]^ A USA observational study,^[29]^ looking at the admissions of over 15,000 patients hospitalized for chronic conditions, also found that longer length of stay was associated with an increased readmission risk (OR: 1.03, 95% CI: 1.02–1.04).

Reducing inpatient admissions, on the other hand, reduces the economic expenditures and improves hospital resource utilization, and hospitals become more efficient at dealing with illnesses.^[30]^ Contrarily, overnight admission may pose a financial burden on the child’s family even though family preferences play a role in bringing a child to the hospital for a certain health event as opposed to home treatment.^[31]^ Many parents often find it difficult to distinguish between serious health conditions and trivial self-limiting illnesses,^[32]^ fear the worst when their child is unwell, and feel uncomfortable taking the child home without an overnight stay in certain cases.^[33]^ Pedro et al found that mothers of children with respiratory diseases possibly have a greater need to take their children to the ED due to their insecure and frightened feelings about the situation.^[34]^ Their emotions can be comprehended since respiratory diseases are found to be the major cause of mortality and morbidity worldwide^[35]^ and is common early in life as infants and young children are especially susceptible. The spectrum of disease ranges from acute infections (lower and upper respiratory tract infections) to chronic non-communicable diseases (asthma). Pneumonia remains the main cause of childhood mortality globally with approximately 1.3 million deaths yearly while asthma is the predominant non-communicable disease in children.^[36]^ Similarly, the findings of the present study found that respiratory disease, such as pneumonia and bronchiolitis, was the leading cause of pediatric admissions (15.1%), followed by injury, poisoning, and certain other consequences of external causes. While respiratory diseases were identified as the primary reason for pediatric hospital admissions in the study, it should be acknowledged that accidents, injuries and other external causes may have higher mortality rates than respiratory illnesses in children in Australia.^[2]^ A recent study in Australia found that chronic obstructive pulmonary disease with acute lower respiratory infection and with acute exacerbation accounted for around 55% of hospital admissions for chronic lower respiratory diseases followed by asthma.^[37]^ Greater independence during childhood, adolescence and young adulthood brings the potential for riskier actions, which can lead to injury. The most common causes of unintentional injury in Australia as reported by Australian Institute of Health and Welfare in 2019, are related to land transport accidents, exposure to inanimate mechanical forces, and falls.^[38]^ Poisoning in Australia is the ninth most common reason for injury hospitalization, and mostly involves pharmaceutical drugs.^[39]^ It is therefore important to consider the implementation of accident prevention programs in order to reduce hospital admissions among adolescents. Given the respiratory illness remains the leading cause of hospitalization in our study, reinforcing vaccination coverage (e.g., influenza and pertussis), promoting early detection, and enhancing parental education on managing respiratory symptoms should be considered priority strategies. Similarly, since injury consistently ranked among the top contributors to admissions, targeted prevention programs such as road safety campaigns, poison-prevention initiatives, and home safety interventions could have direct impact.^[40–44]^ These evidence-based measures align with the main findings of our study and highlight actionable opportunities for reducing pediatric hospitalization rates in Australia. Diseases of the digestive system was ranked the third at 12.1%. The most reported Gastrointestinal disorders among hospitalized children are acute diarrheal diseases, appendicle conditions, abdominal pain, esophageal disorders, and digestive congenital anomalies.^[45]^

The present study found growing numbers (increase by 89.7%) of pediatric patients with neurological conditions being admitted to hospitals in Australia. In England, between 2004 and 2014, there was also a significant increase (51%) in the number of children with a neurological condition.^[46]^ In the USA, the proportion of hospitalizations attributable to children with neurological impairments also increased from 7.3% in 1997 to 9.9% in 2006.^[47]^ Similar data have been reported from the EU28 and WHO European region among adults.^[48]^ The increased hospital admissions by children with neurological conditions in our study and in previous studies^[46,47]^ emphasizes the importance of recruitment and retention of clinical pediatric neurological specialists or to work in collaboration with GPs to provide the best possible care.

The present study also shows an increase of 57.4% in hospital admission rates among pediatrics with diseases of the circulatory system. Children with congenital heart disease (CHD) are at a higher risk of requiring hospitalization as observed through a study that found CHD-related ED visits to have higher rates of inpatient admission and a higher mortality rate compared to non-CHD related visits.^[49]^ Hemmo et al also found a higher hospital admission rate due to ischemic heart disease among children below 15 years in England and Wales (a 114.5% increase from 0.28 [95% CI = 0.18–0.39] in 1999 to 0.61 [95% CI = 0.46–0.75] in 2019 per 100,000 persons).^[50]^ These findings highlight the importance of early detection and management of circulatory diseases in pediatric patients to prevent adverse outcomes and reduce the burden on healthcare services.

Interestingly, the considerable changes in lifestyle and dietary habits observed in children, such as decreased physical activity and increased consumption of energy-dense foods and sugary drinks, are not only linked to cardiovascular disorders, but also to endocrine, nutritional, and metabolic ailments. A substantial increase of type 2 diabetes in children and adolescents was reported with approximately 41,600 new cases of diagnosed type 2 diabetes in 2021 worldwide.^[51]^ Additionally, the prevalence of overweight and obesity has dramatically increased from 4% in 1975 to over 18% in 2016 among children and adolescents aged 5 to 19 years,^[52]^ resulting in a sustained rise in the prevalence of type 2 diabetes mellitus, other endocrine, and nutritional diseases manifested in this study.

On the other hand, the hospital admission rates among pediatrics for pregnancy, childbirth, and the puerperium has more than halved since 1998. This decline can be attributed to the decrease in the proportion of teenage mothers, which has decreased from 3.8% in 2010 to 1.8% in 2020^[53]^ and it is probably due to effective sexual health education and ease of access to contraception, rather than any decrease in sexual activity. Mark and Wu have recently demonstrated that increased funding for comprehensive sex education in the USA has led to a reduction in the teen birth rate by over 3% at the county level^[54]^ and undoubtedly, increased access to contraception has played a crucial role in reducing adolescent pregnancies and births, as evidenced by the significant decreases in rates of teenage pregnancy and childbearing in recent years.^[55]^

Our finding of higher admission episodes of male pediatrics (53.3%) is consistent with prior research.^[56]^ The preponderance of males on admission could be due to injury and poisoning which contributed to almost a quarter (23%) of the reasons for hospitalization of males aged 15 to 24. Interestingly, female admissions rose markedly (by 6.1%) over the study period compared to male children (rose by 0.1%). Although our data did not allow us to determine the precise cause of the observed rise in female pediatric admissions, prior research has suggested potential explanations. A previous study identified that children with a mental disorder had a higher risk of hospitalization than their peers and mental and behavioral disorders accounted for higher readmission rate (28.3%) for females than for males (20.3%) who were hospitalized with a mental disorder.^[57]^ Furthermore, female children with a mental disorder had a higher proportion (14.3%) of co-occurring mental disorders than males (10.2%).^[58]^ Peripartum psychiatric disorders among teenage mothers could explain the upward trend since it was noted by Egorova et al that 68 of every 1000 admissions for delivery among US females ages 12 to 20 had 1 or more behavioral disorders.^[59]^

The admission to hospital for female children has risen not only because of mental and behavioral disorders but also because of nutritional and metabolic diseases noted over the study time. Thyroid diseases, for instance, are 5 to 8 times more common in females than in males.^[58]^ Previous evidence confirms a greater insulin resistance in girls than in boys during puberty and thus a higher prevalence of type 2 diabetes mellitus in adolescent females than males.^[60]^ Moreover, diabetic ketoacidosis incidence rate is found to be significantly higher in females.^[61–63]^ As a result, hospitalization due to nutritional and metabolic disorders is common among female patients.^[64,65]^

The peak age of hospital admission was found to be 0 to 4 years, followed by the age group 15 to 19 years. Children are admitted up to 8 times in the 1st year of life and up to 12 times in the 1st 7 years of life.^[9]^ It is not surprising that hospital admission rates are higher among children aged 0 to 4 years, given that this age group has an immature immune system^[66]^ and is more likely to be exposed to infectious diseases in settings such as day-care or preschool.^[67]^ Additionally, children in this age range are still developing physically and mentally, which increases their risk of accidents and injuries.^[68]^ Furthermore, having underlying diseases along with their limited communication skills can make it challenging to detect and address health issues before they escalate.

Use of age-specific admission rates for children in the present study showed that a decrease in admission over the period 1998 to 2019 only existed within the 1st 4 years while the biggest increase was seen in 15 to 19-year category. The admission rate for children 5 to 9 years remained relatively stable over the period.

Reducing the rate of hospital admissions among infants by 2.5% could be elucidated by the improvements in early detection and screening programs, better diagnostic procedures, and the vaccination strategy. Reviewing 22 model-based studies, it was projected that maternal vaccination and direct infant immunization could potentially result in a 27% and 50% reduction, respectively, in hospitalizations of infants due to respiratory syncytial virus.^[69]^ Moreover, early detection of neonatal abnormalities allows for informed decision-making and can also help in reducing the risk of having abnormalities in newborns.^[70]^ On the other hand, since the diagnosis and prognosis of most chronic childhood conditions have improved considerably in recent decades.^[71]^ There has presumably been an increase in the number of older children and adolescents living with a chronic disease and, therefore, more hospital admissions.^[72]^ For example, mental health disorders during childhood and adolescence, which have gained significant attention in recent times, may have lasting effects into adulthood and could even lead to hospital admission in severe cases.^[73]^

The reasons why children were admitted to the hospital varied by age group as reported in the literature and were consistent with our findings. Hospital admission of children under 1 year of age was attributed by certain infectious and parasitic diseases, diseases of the respiratory system, certain conditions originating in the perinatal period, while injury and poisoning, diseases of the digestive system, and mental and behavioral disorders were the main reason of hospitalization among older children and adolescents.^[74]^ Adolescents may engage in risky behaviors or participate in sports activities that increase their risk of injury or poisoning. In addition, they may consume unhealthy diets, such as foods high in fat and sugar, which can lead to digestive problems. Adolescents may also experience stress and mental health problems related to school performance, peer relationships, family issues, and other factors. This can lead to depression, anxiety, and eating disorders, which may require hospitalization.

One limitation of this study is that patients who are over 16 years old are typically not treated by pediatric clinicians, as they are considered to have entered adulthood. As a result, the observed increase in admission rates among the 15 to 19 age group may not accurately reflect a true increase. The use of aggregated data on population-level restricted our ability to identify other important confounding factors that might have influenced the admission rate. Additional limitations include the possibility of coding drift over time, which may affect comparability of diagnostic categories; the inability to account for multiple admissions per child (readmissions); the lack of socioeconomic or indigenous status stratification, which are known to influence pediatric health outcomes. Our dataset represents hospital admission episodes rather than unique patients; repeated admissions by the same individual may have contributed to overall counts, potentially inflating the burden of some conditions.

## 5. Conclusion

Overall hospitalization rate for pediatric patients remained largely stable during the past 2 decades. Important shifts were observed, including a rise in same-day admissions and higher admission rates among females. The predominance of respiratory conditions in young children and increasing hospitalizations in adolescents, particularly females, highlight the need for targeted strategies such as improved respiratory infection prevention, early parental guidance for acute illness, injury prevention programs, and expanded youth mental health services.

## Acknowledgments

We would like to acknowledge Princess Nourah bint Abdulrahman University Researchers Supporting Project number (PNURSP2026R483), Princess Nourah bint Abdulrahman University, Riyadh, Saudi Arabia.

## Author contributions

**Conceptualization:** Esra’ O. Taybeh, Alaa A. Alsharif, Abdallah Y. Naser.

**Data curation:** Abdallah Y. Naser.

**Formal analysis:** Abdallah Y. Naser.

**Funding acquisition:** Alaa A. Alsharif, Abdallah Y. Naser.

**Investigation:** Esra’ O. Taybeh, Alaa A. Alsharif, Abdallah Y. Naser.

**Methodology:** Abdallah Y. Naser.

**Project administration:** Abdallah Y. Naser.

**Resources:** Esra’ O. Taybeh, Abdallah Y. Naser.

**Software:** Abdallah Y. Naser.

**Supervision:** Esra’ O. Taybeh, Abdallah Y. Naser.

**Validation:** Esra’ O. Taybeh, Alaa A. Alsharif, Abdallah Y. Naser.

**Visualization:** Esra’ O. Taybeh, Alaa A. Alsharif, Abdallah Y. Naser.

**Writing – original draft:** Esra’ O. Taybeh, Alaa A. Alsharif, Abdallah Y. Naser.

**Writing – review & editing:** Esra’ O. Taybeh, Alaa A. Alsharif, Abdallah Y. Naser.

## Supplementary Material
