# Supplementary material for: Pediatric hospitalization profile in Australia: A longitudinal ecological study, 1998 to 2019
Source: Medicine (Baltimore). 2026 Mar 13;105(11):e47986. doi: 10.1097/MD.0000000000047986 (PMC12991756; doi:10.1097/MD.0000000000047986)

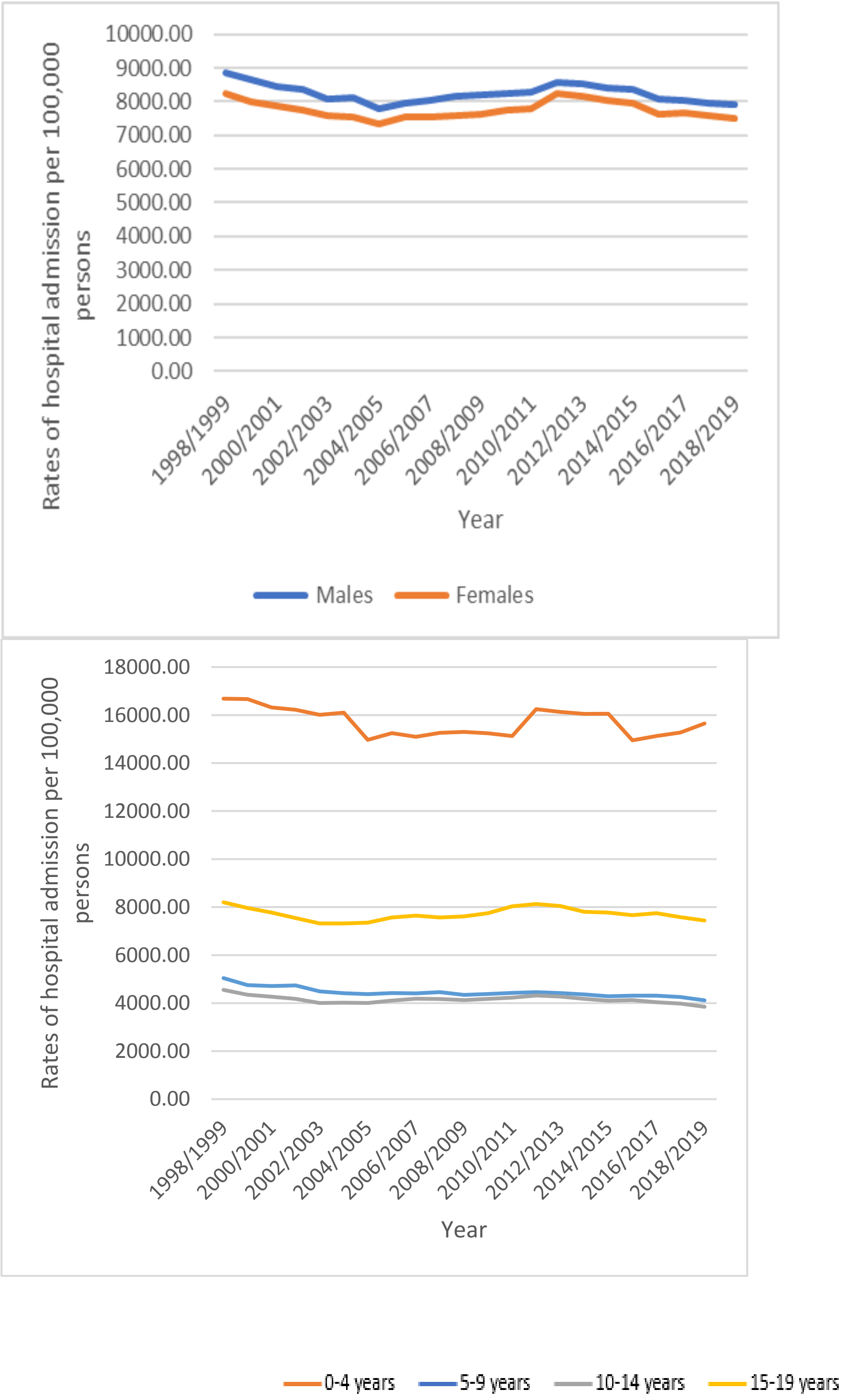

Figure **S1** Rates of overnight hospital admission among pediatrics in Australia stratified by gender and age

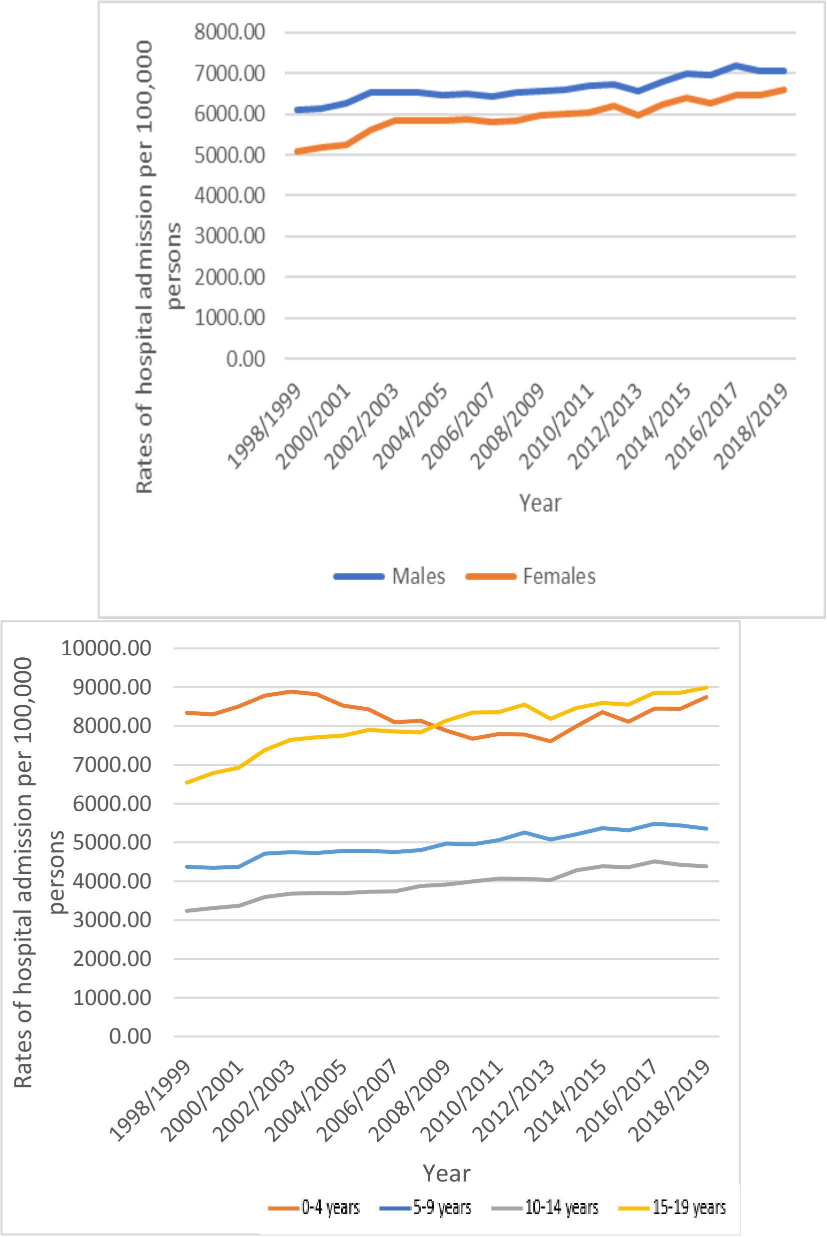

Figure **S2** Rates of same-day hospital admission among pediatrics in Australia stratified by gender and age

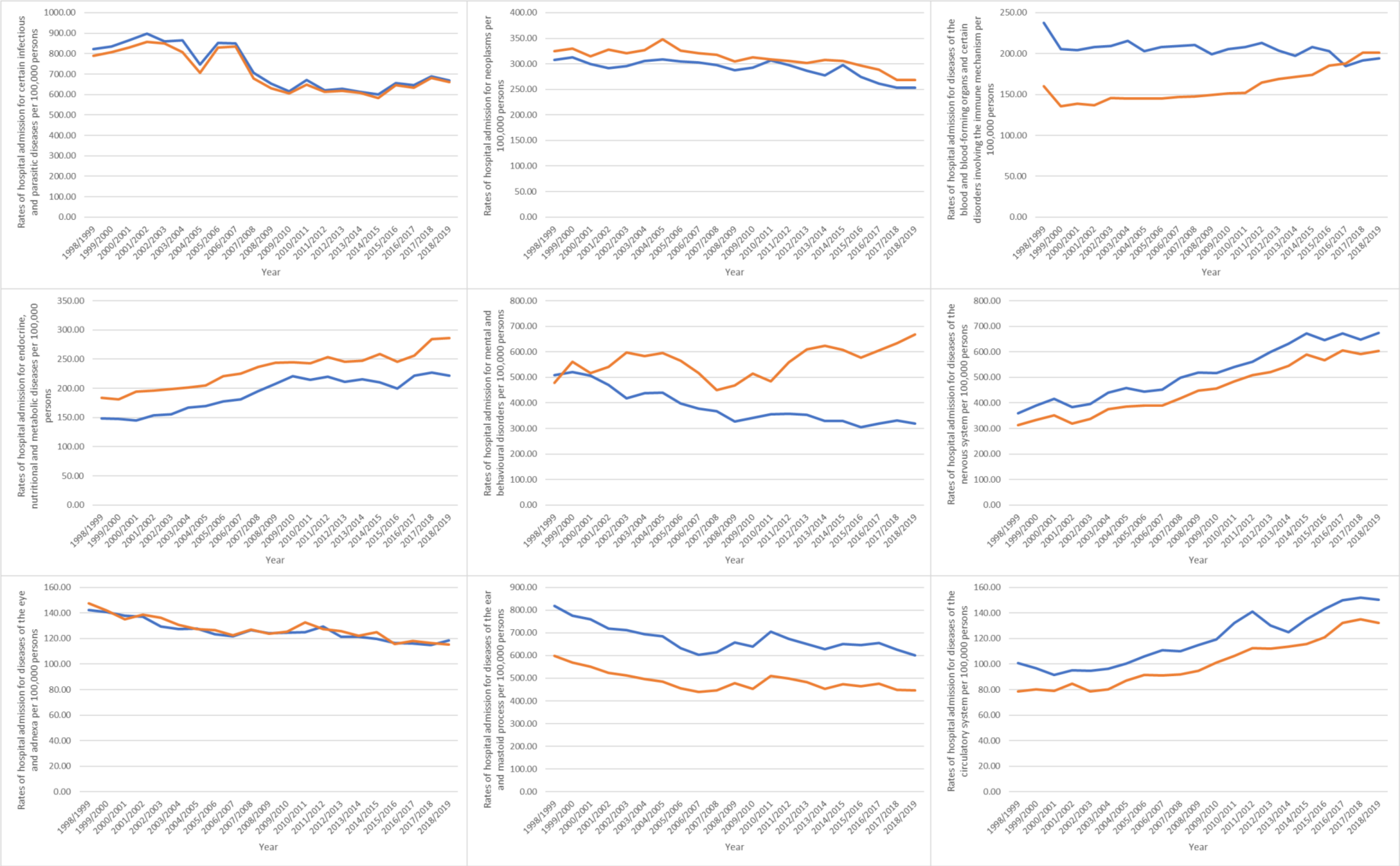

Figure S3 (A) Rates of hospital admission among pediatrics in Australia stratified by gender between

Males Females

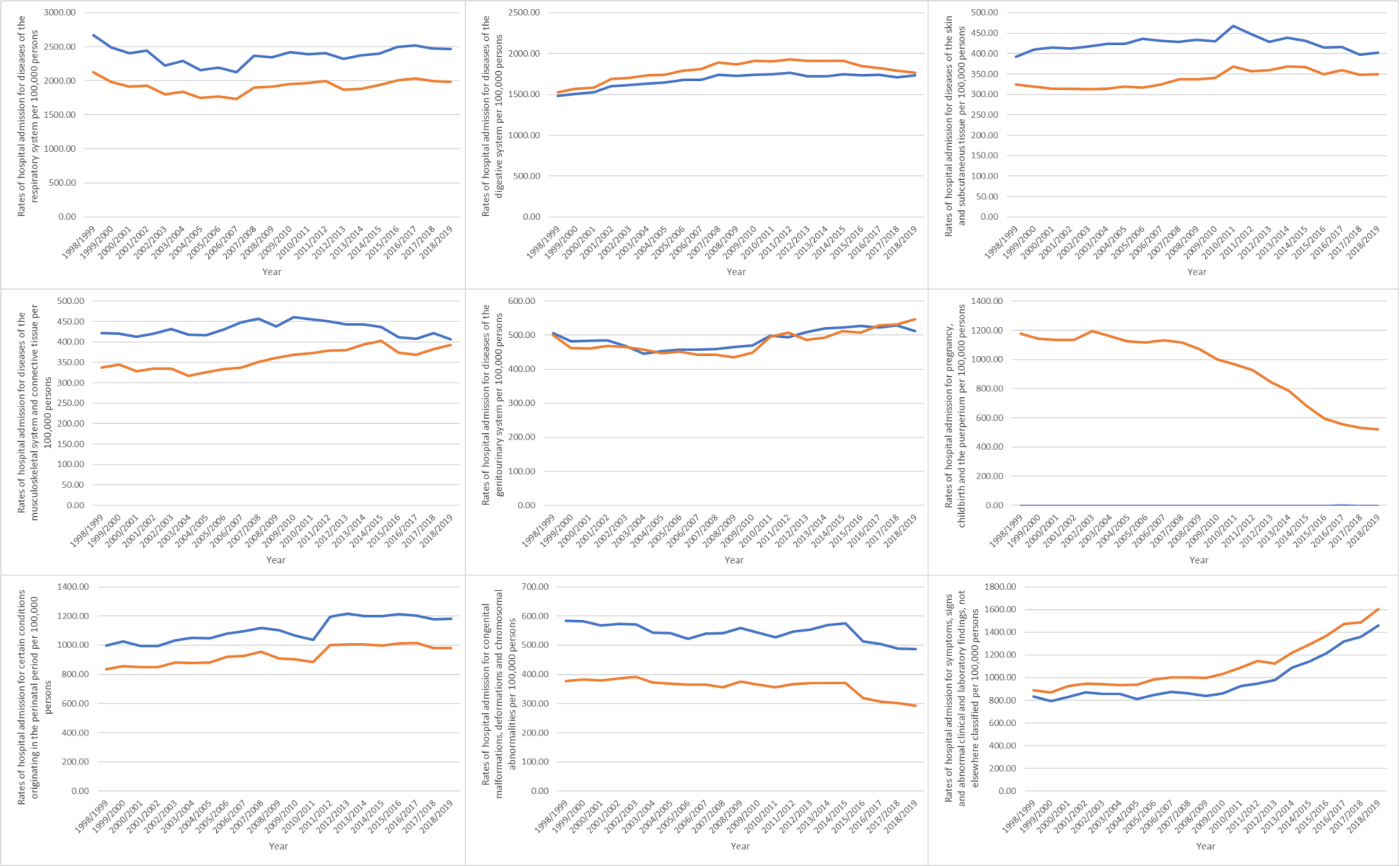

Figure S3 (B) Rates of hospital admission among pediatrics in Australia stratified by gender

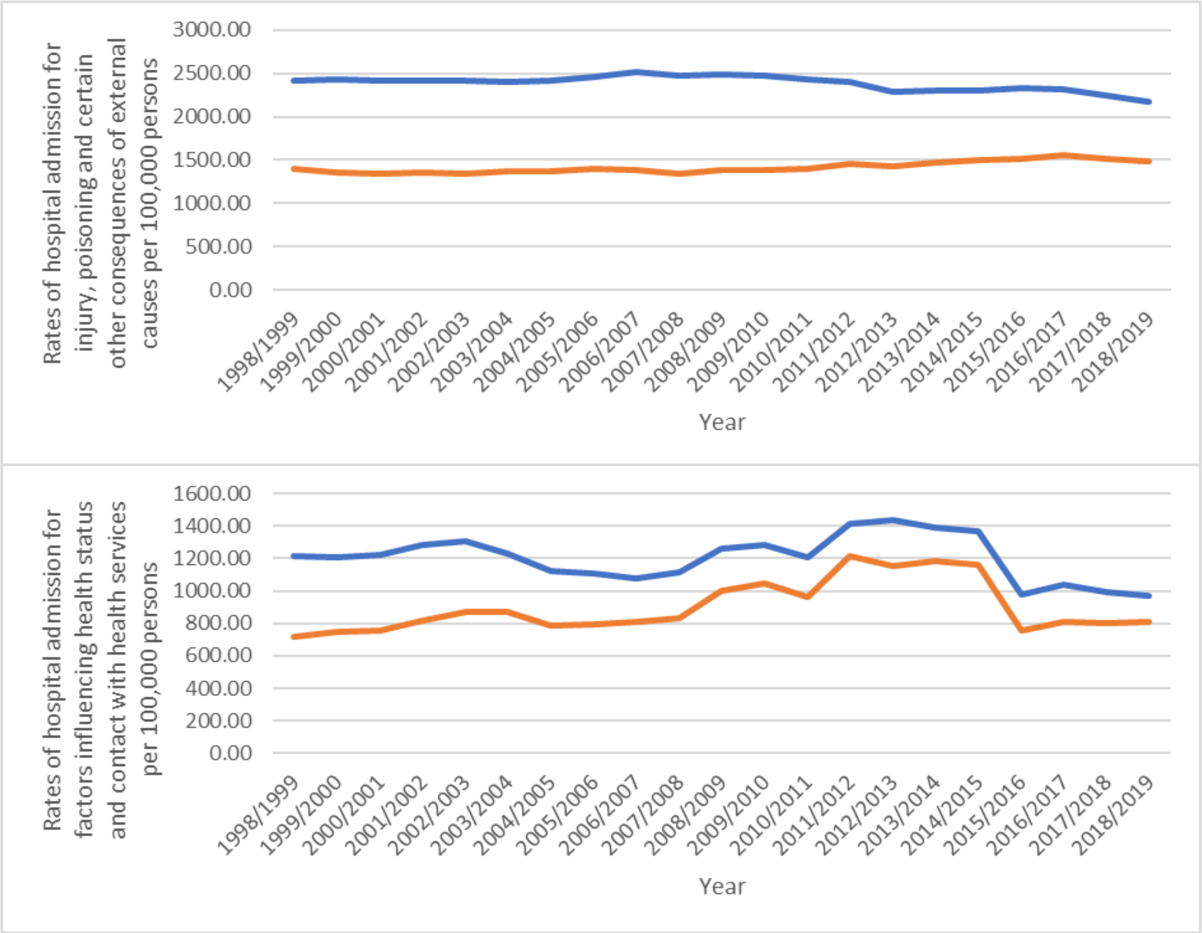

Figure S3 (C) Rates of hospital admission among pediatrics in Australia stratified by gender between 1998 and 2019.

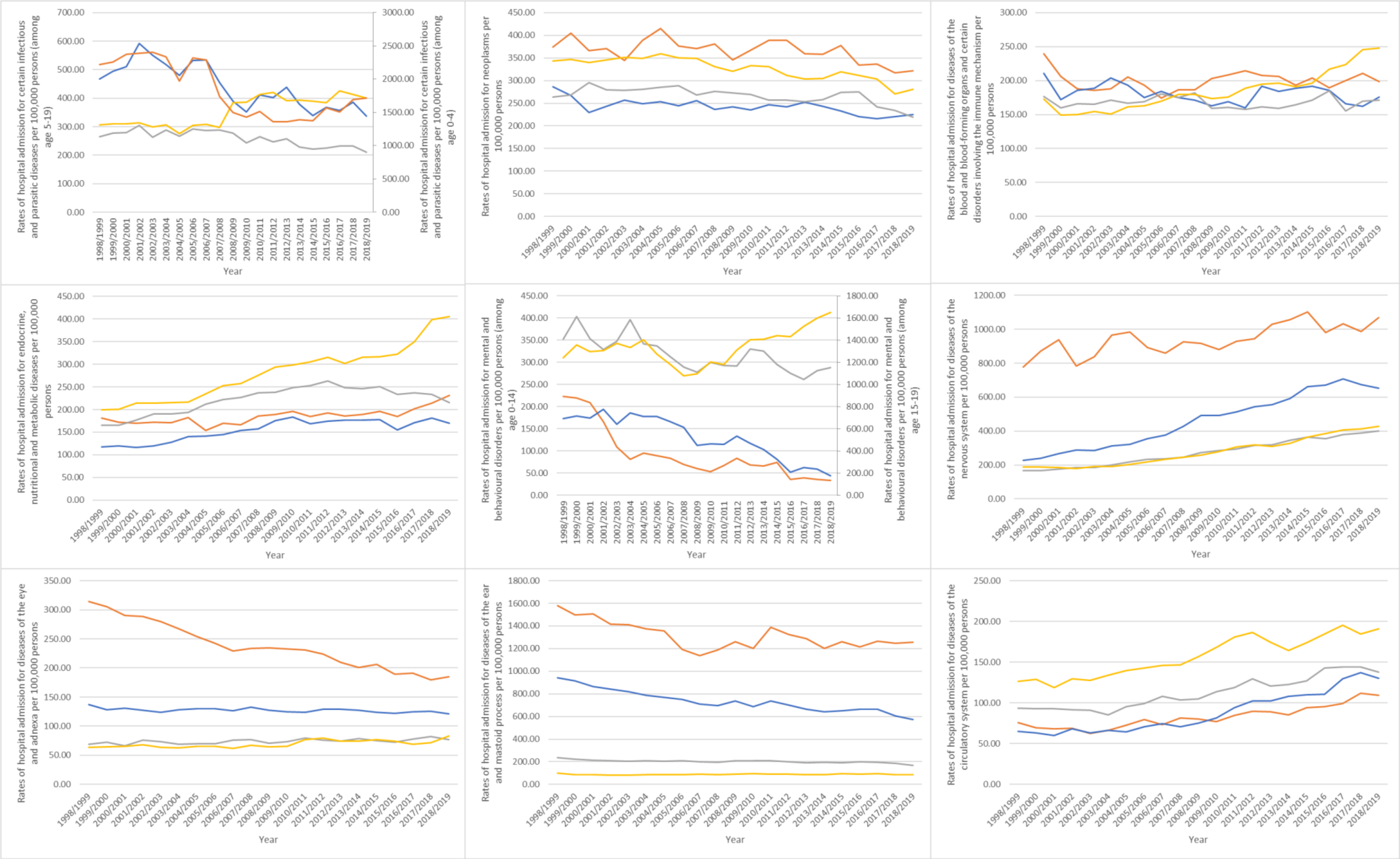

Figure **S4** (A) Rates of hospital admission among pediatrics in Australia stratified by age group between

0-4 years   5-9 years   10-14 years   15-19 years

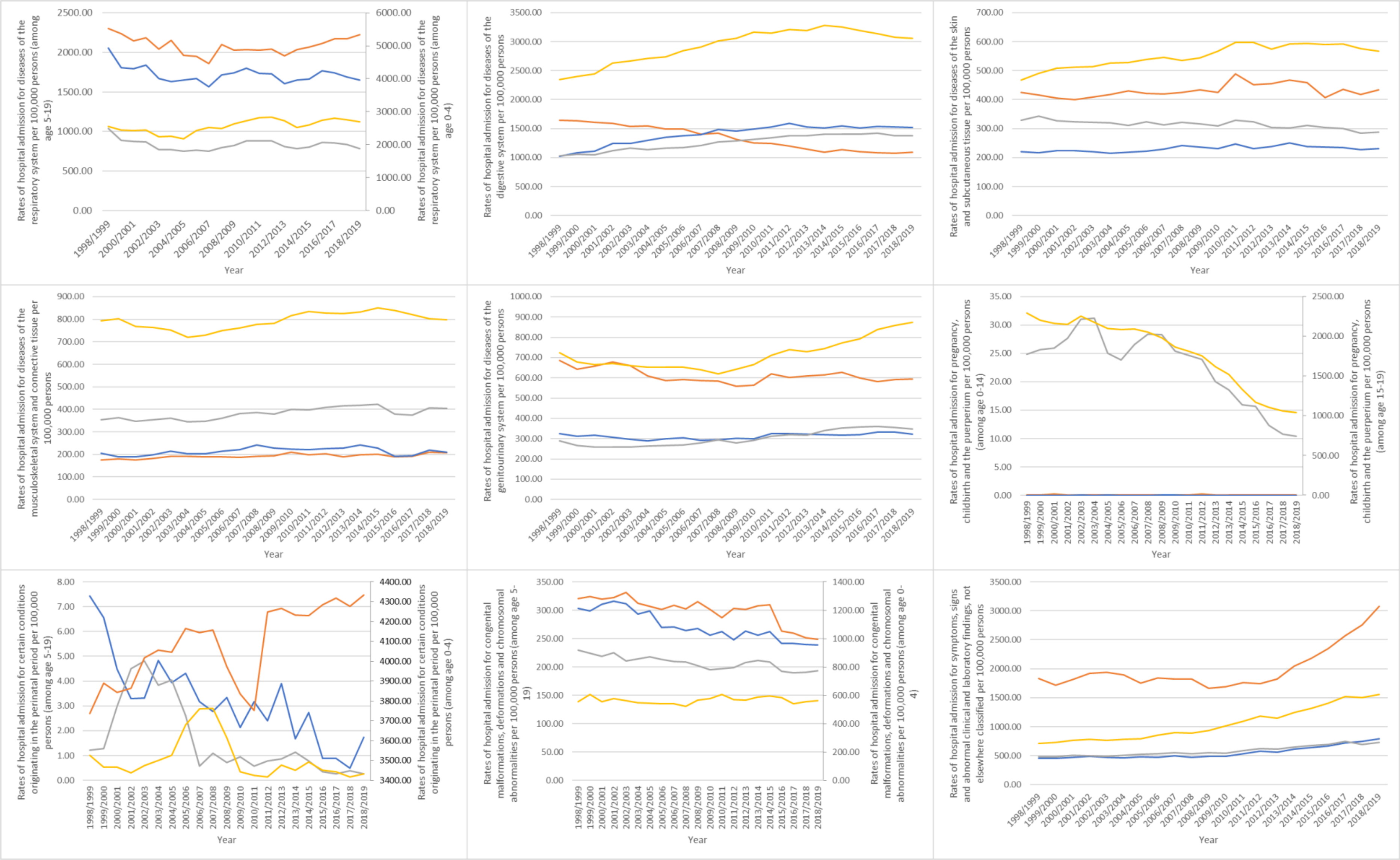

Figure **S4 (B)** Rates of hospital admission among pediatrics in Australia stratified by age group between

0-4 years 5-9 years 10-14 years 15-19 years

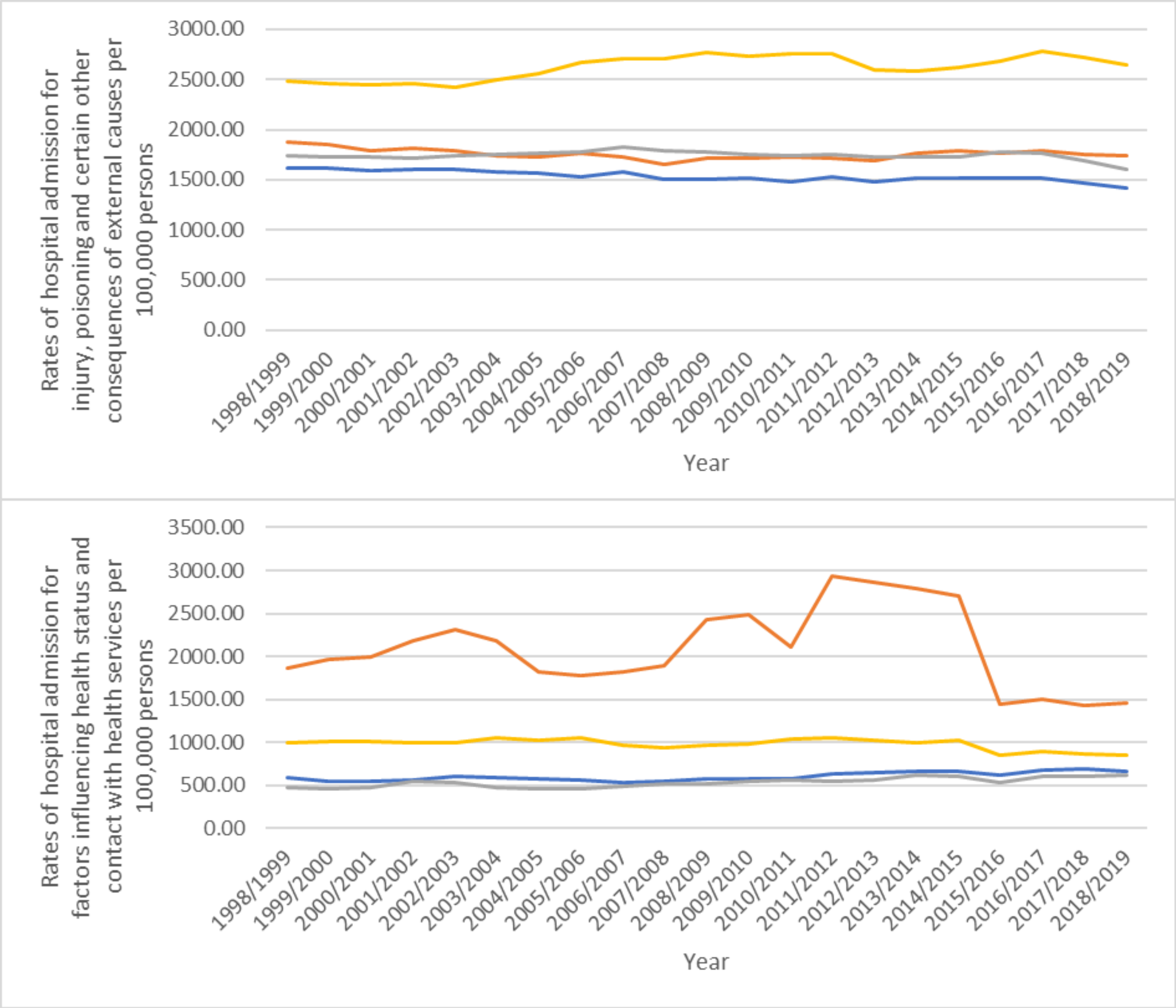

Figure S4 (C) Rates of hospital admission among pediatrics in Australia stratified by age group between 1998 and 2019.

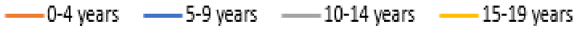

Supplement: Supplementary file 1 [file medi-105-e47986-s001.pdf]
